# Solar-Driven Photocatalytic Films: Synthesis Approaches, Factors Affecting Environmental Activity, and Characterization Features

**DOI:** 10.1007/s41061-022-00409-2

**Published:** 2022-10-01

**Authors:** Andraž Šuligoj, Romana Cerc Korošec, Gregor Žerjav, Nataša Novak Tušar, Urška Lavrenčič Štangar

**Affiliations:** 1 Faculty of Chemistry and Chemical Technology, University of Ljubljana, Večna pot 113, 1000 Ljubljana, Slovenia; 2 National Institute of Chemistry, Hajdrihova 19, 1000 Ljubljana, Slovenia; 3 Graduate School, University of Nova Gorica, Vipavska 13, 5000 Nova Gorica, Slovenia

**Keywords:** Degradation, Efficiency, Thickness, Structure, Characterization

## Abstract

Solar-powered photocatalysis has come a long way since its humble beginnings in the 1990s, producing more than a thousand research papers per year over the past decade. In this review, immobilized photocatalysts operating under sunlight are highlighted. First, a literature review of solar-driven films is presented, along with some fundamental operational differences in relation to reactions involving suspended nanoparticles. Common strategies for achieving sunlight activity from films are then described, including doping, surface grafting, semiconductor coupling, and defect engineering. Synthetic routes to fabricate photocatalytically active films are briefly reviewed, followed by the important factors that determine solar photocatalysis efficiency, such as film thickness and structure. Finally, some important and specific characterization methods for films are described. This review shows that there are two main challenges in the study of photocatalytic materials in the form of (thin) films. First, the production of stable and efficient solar-driven films is still a challenge that requires an integrated approach from synthesis to characterization. The second is the difficulty in properly characterizing films. In any case, the research community needs to address these, as solar-driven photocatalytic films represent a viable option for sustainable air and water purification.

## Introduction

**Fig. 1:**
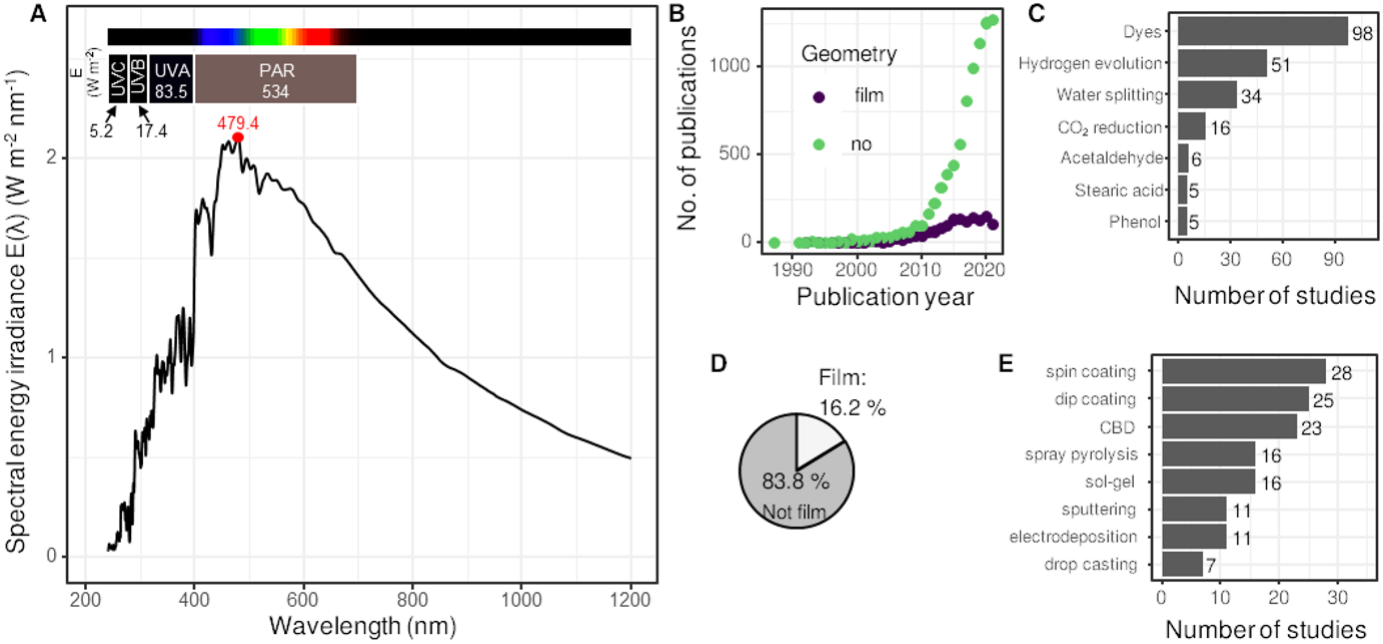
(**A**) The solar spectrum at the ground level in Colorado, USA, on 25 June 2020, with calculated irradiance levels from the UVC to PAR range. Statistics on a web search for the search term “solar photocatalyst” in titles and abstracts (Web of Science [WOS], January 2022): **B** the number of publications per year, **C** the most common target reactions when films are used, **D** the share of publications that explicitly contain the words “film” or “layer” in the title or abstract, and **E** the most prominent deposition methods

Solar photocatalysis, a part of the broader concept of advanced oxidation processes (AOPs) that also includes other technologies such as Fenton, ozonation, and photocatalysis in general, is one of the most widely applied technologies in its field. The commercial aspects of solar photocatalysis go back to the mid-1990s in Japan and spread later also to North America and Europe [1]. As such, it is one of the most interesting fields, probably due to its environmentally friendly nature and the fact that it promises to employ solar irradiation as its energy source, a source which is bountiful in many parts of the Earth. We thus first define the term “solar.” This term encompasses not only the visible part of the solar spectrum (sometimes also defined as photosynthetically active radiation, PAR)—as the terms solar and visible are commonly used as synonyms—but also a portion of the ultraviolet (UV) (UV-A, UV-B, as well as UV-C) (Fig. 1A). In addition, the solar irradiance, as measured at ground level, contains a rather large portion of near infrared (IR) irradiation. This is important because, as we will see later, some researchers have focused on this part of the spectrum for enhancement of the photocatalytic activity of their materials [2].

Immobilization of photocatalysts is an efficient way to reduce the potential negative effects caused by the release of nanoparticles (NPs) into the environment. Immobilization can lead to many macroforms such as pellets, spheres, films, macroaggregates, etc. Herein, we focus on studies of solar photocatalysts that are immobilized in film form, regardless of the curvature/roughness of the underlying support, but omit publications in which the photocatalyst is immobilized in other forms, such as floating macroaggregates [3]. We thus need to define the term “film.” According to the International Union of Pure and Applied Chemistry (IUPAC) [4], this term signifies condensed matter restricted to one dimension, while the term “thin film” stands for a film whose thickness is on the order of a characteristic scale or smaller; For example, a film may appear thin or thick operationally, according to the procedure applied. Hence, it is also recommended that the measurement procedure employed be specified (e.g., ellipsometrically thin film, optically thin film, etc.) [4]. As described in the following sections, researchers often define their system as thin even when the thickness might measure several microns. These are typical examples of optically thin films [5]. The lack of consistency and inadequate indication of thickness is obvious (as shown below). We thus focus here on macroscopic films/coatings, whether thin or thick, but refrain from discussing the works in which the immobilization of the material on the underlying substrate also leads to the use of the word “film” even when it stands for nanomodified material used in reactions in powder form [6].

The advantages of substituting powder dispersion with photocatalytic films are summarized in Table 1. However, the share of publications on solar photocatalysis in immobilized form of photocatalyst—films or coatings—barely surpasses 16% (Fig. 1D). Nevertheless, this share cannot be neglected. Noteworthily, a similar share for films (15%) is also found in the corpus of patents obtained from the Google patents site (not shown here, using the search term “solar photocatalyst,” yielding 25,000 results). The importance of films can furthermore be seen through past publications. Arguably the most influential paper on photocatalysis was published in 1972 by Fujishima and Honda, describing work conducted on 1.5-mm-thick titanium dioxide (TiO_2_) rutile wafer [7]. Additionally, one of the most influential papers on a visible-light active photocatalyst, viz. N-doped TiO_2_, was also conducted on titania film [8]. Doping improved the TiO_2_ visible light optical absorption substantially and consequentially the photocatalytic activity. Films become especially important when solar photocatalysis in flow mode is considered. The reader is referred to a more in-depth review on this subject elsewhere [9].

**Table 1:** Comparison between powder/colloidal and immobilized photocatalytic systems

Slurry system	Supported system
*Advantages*	
Good surface-to-volume ratio	No particle–fluid separation needed
No mass-transfer limitations	Films can be immobilized on UV-transparent substrates
	Improved fluid dynamics of reactor
	Flexibility in reactor design
	Configurations in which all the catalyst is illuminated are possible
*Disadvantages*	
Particle–fluid separation	Reduction of active surface area
Rigid reactor design	Mass-transfer limitations
Limited penetration depth of light	Presence of foreign species (Na^+^, Si^4+^, Fe^3+^, etc.)
	Difficulty of designing stable coatings

When evaluating the advantages and disadvantages of using films as opposed to powders, it is also important to summarize what governs the photocatalytic activity of a semiconductor. The three fundamental parameters are: (1) the light absorption properties of the material, (2) the rate of reduction and oxidation of the adsorbed molecule by the charge carriers, and (3) the rate of recombination of electron–hole pairs. In solar light applications, one must additionally consider both light absorption as well as light scattering, although the latter is often neglected in literature. It is well known that increasing the amount of powder in suspension eventually leads to saturation of absorption while a further increase in the powder content has a detrimental effect on the system kinetics. This is mainly due to increased scattering, leading to lower absorption, and increased charge recombination. Maintaining comparable/optimal light absorption by all particles in a catalyst system at high volumes of liquid reactants is a major challenge, and this is unlikely to be achieved, especially with sunlight [5, 10]. Therefore, limiting the photocatalyst to two dimensions and carefully adjusting the aggregation of nanoparticles can lead to high efficiencies and low catalyst consumption, as well as long-term stability of the system. In addition, proper contact of the active photocatalysts (together with binders, if necessary) on the solid substrate enables efficient mass transport of reactants, intermediates, and products. This is especially true for systems such as dye-sensitized solar cells (DSSCs), where the transport of charge carriers is one of the most important factors limiting the efficiency of the cell [11, 12].

**Fig. 2:**
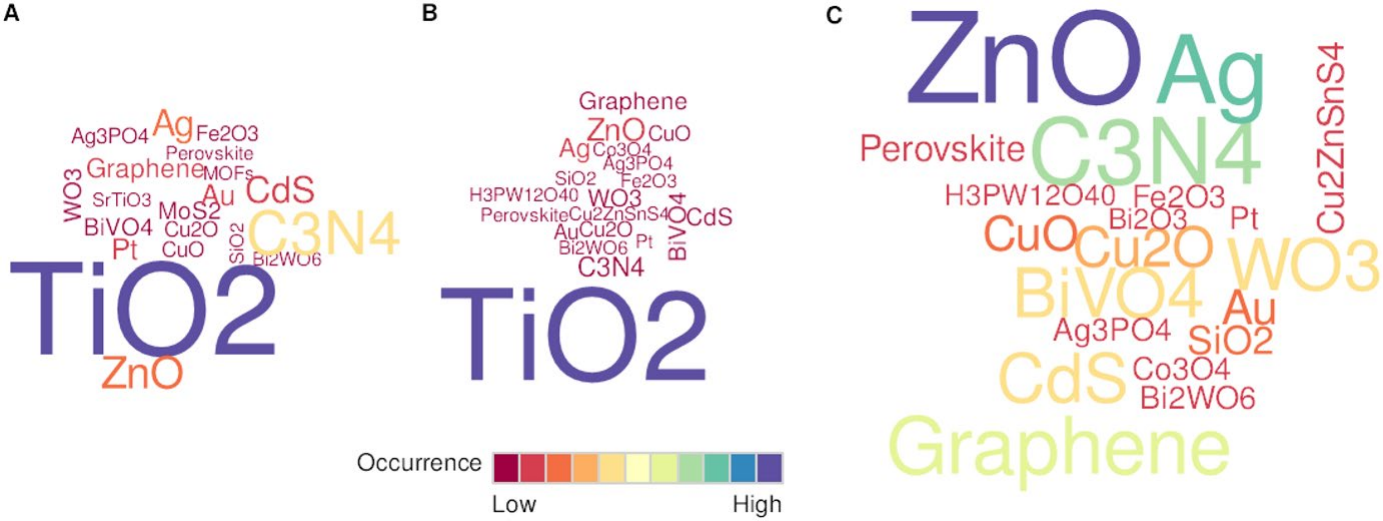
Word cloud of the most prominent materials found in abstracts in the researched literature (WOS, search term “solar photocatalyst”, no. of results 9000), showing the most prevalent materials in **A** the whole corpus, **B** papers dealing with immobilized systems, and **C** regarding immobilized systems excluding TiO_2_

A close look at the target reaction systems reveals that the majority are reactions done with dyes, followed by hydrogen evolution, water oxidation, and CO_2_ reduction (Fig. 1C). Next, we examined the authors’ keywords from the corpus of publications obtained from the Web of Science (WOS) database and split the publications into those dealing with films and those not (Fig. 2). Interestingly, the topics largely coincide, with a few exceptions. The top four keywords (i.e., “photocatalysis,” “TiO_2_,” “water splitting,” and “visible light”) appear in virtually the same order. However, in articles dealing with films, the term “photocatalytic activity” is much more common, while the topic of hydrogen evolution is less prevalent. This shows that reactions in film form mainly focused on degradation (keywords analysis) with most systems being dyes (abstract analysis). Regarding the materials used, TiO_2_ is by far the most widely studied, in both immobilized as well as powder form (Fig. 2), although it suffers from some drawbacks such as (1) a wide bandgap energy of 3.0–3.2 eV (*λ* < 387 nm) and (2) a fast electron–hole recombination rate [13, 14]. The other materials found commonly in literature are zinc oxide, graphitic carbon nitride, graphene, silver, cadmium sulfide, and others.

In what follows, we describe the important factors that determine the efficiency of solar photocatalysis when used in immobilized, film form. The focus is on degradation reactions as they are recognized to be the most prominent reaction systems (Fig. 1C). We then describe the characterization techniques that are or should be used by researchers to properly define each photoactive system. Finally, we present conclusions and an outlook on the field.

## Active Species in Film Solar Photocatalysis

**Fig. 3:**
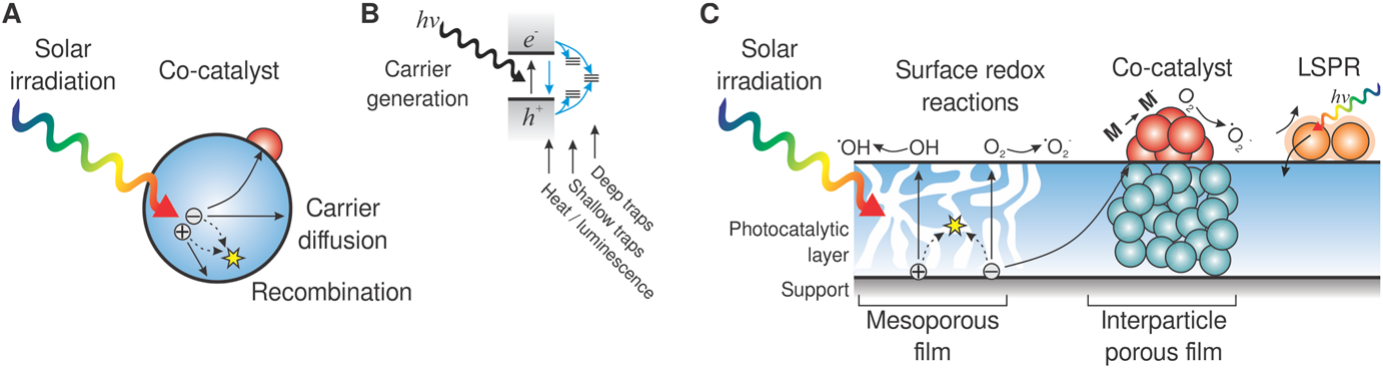
Schematic of photocatalytic reaction phenomena in **A** nanoparticle, **B** photoproduction and intraparticle fate of charge carriers, and **C** depiction of selected reactions occurring in/on films. LSPR, localized surface plasmon resonance

As mentioned above, the utilization of charge carriers is one of the three key parameters for efficient and highly active photocatalytic materials. The main components responsible for sunlight activity are the same for powder/colloidal and immobilized systems. Although the detailed mechanisms of the photocatalytic reactions vary depending on the nature of the pollutants, there is general agreement that the primary reactions responsible for the photocatalytic effect are the redox reactions of the pollutants at the interface with the charge carriers of the photocatalyst when irradiated with light of sufficient energy (Fig. 3). A detailed overview of the reactive oxygen species (ROS) involved in photocatalysis, also touching on the reactions triggered by sunlight irradiation, is given elsewhere [15]. The charge carriers can be used directly, i.e., through oxidation with holes (h^+^) or reduction with electrons (e^–^). However, these charge carriers usually react with adsorbed species on the surface to form other, more mobile reactive radicals (Fig. 3C).

It has been pointed out that the photogenerated oxidizing species do not migrate far from the photogenerated active sites and that the degradation process occurs at the surface of or within a few monolayers around the photocatalytic particles (Fig. 3) [16]. This fact is supported by the timescales of the primary photocatalytic processes. For example, the generation of charge carriers in the case of titanium dioxide (TiO_2_ + *hν* → e^–^ + h^+^) occurs within 1 fs, the trapping of charges at the surface (h^+^  → h_tr_^+^, e^–^ → e_tr_^–^) occurs in the range of 100–200 fs, the recombination in the bulk of the photocatalyst (h^+^  + e^–^, h^+^_tr_ + e_tr_^–^ → heat (or *hν*)) occurs in 1 µs (water) or 25 µs (air), and the oxidation of organics by trapped holes takes from 300 ps for methanol to 2 µs for water, while the reaction of O_2_ with trapped e^–^ occurs in < 100 ns [16]. Therefore, it is of great importance to identify the oxidizing species formed on the surface of the irradiated photocatalysts. These species in solar photocatalysis include free or trapped holes, OH radicals (^**·**^OH), superoxide anions (O_2_^**·**–^), and singlet oxygen (^1^O_2_). O_2_ and H_2_O_2_ are also involved in oxidation reactions in various mechanisms. Because of their paramount importance in photocatalytic reactions, we devote a brief section to these species here, focusing on those that occur under sunlight irradiation.

### Superoxide Radicals

**Fig. 4:**
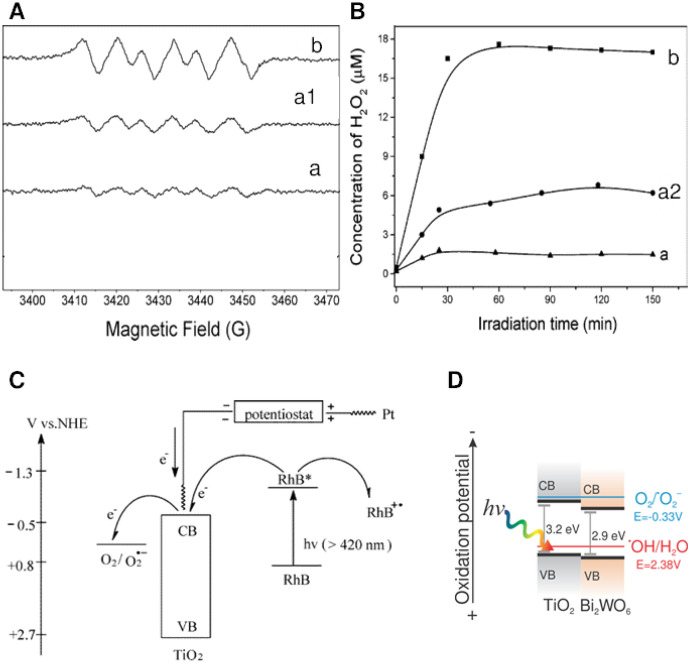
ESR spectra for superoxide radical (^**·**^O_2_^–^/HOO^**·**^) adducts with DMPO during photodegradation of Rhodamine B (RhB) (**A**), the concentration profiles for the formation of H_2_O_2_ during the degradation of RhB (10 μM) (**B**), the proposed mechanism of the degradation of RhB under visible-light irradiation (**C**), and example VB and CB positions for TiO_2_ and Bi_2_WO_6_ and the potential of the radicals’ redox couples ^**·**^OH/H_2_O and O_2_/^**·**^O_2_^−^. In ESR spectra, (a) shows 0.6 V versus saturated calomel electrode (SCE) under visible irradiation; (a1) −0.6 V versus SCE in the dark; (b) −0.6 V versus SCE under visible irradiation. In H_2_O_2_ concentration profiles, (a) shows profile of 0.6 V versus SCE under visible irradiation; (a2) with only electrolysis at a bias of −0.6 V versus SCE; (b) under visible light and at a bias of −0.6 V versus SCE [22] Reproduced with permission from the American Chemical Society

When reducing surface-bound O_2_, one can expect the formation of active oxygen species such as ^·^O_2_^–^ (or HOO^**·**^ in acidic media) and H_2_O_2_. Superoxide radicals have been proven to be the species responsible for visible light activity by many groups in many different semiconductors: TiO_2_ [17], g-C_3_N_4_ [18], and also composite materials [19, 20]. Regardless of the semiconductor used, the formation of ^**·**^O_2_^–^ is commonly followed by 5,5-dimethyl-1-pyrroline-*N*-oxide (DMPO) spin-trapping electron spin resonance (ESR) techniques (Fig. 4A), or, more simply, by quenching reactions with *p*-benzoquinone. Redox potentials of oxygen species change by −0.059pH (at 25 °C) along with pH change according to the Nernst equation for the release of one proton. ^**·**^O_2_^–^ is one exception to this rule in the pH range of 4.8–11.9, due to the involvement of two protons in its reduction: ^**·**^O_2_^−^  + 2H^+^  + e^−^  → H_2_O_2_. Thus, dioxygen can easily trap an electron from the conduction band of a semiconductor with conduction-band potentials more negative than the reduction potential of O_2_ (−0.33 V versus normal hydrogen electrode (NHE), O_2_/^**·**^O_2_^−^); for example, ^**·**^O_2_^–^ was found to be responsible for visible-light bisphenol-A (BPA) degradation via hydroxylation of BPA by ^**·**^O_2_^–^ [21], or photoelectrocatalytical degradation of cationic dyes by films (TiO_2_) [22].

### Hydroxyl Radicals

Hydroxyl radicals (^**·**^OH) are arguably the most sought-after oxidative species in photocatalysis, due to their nonselectivity and high oxidation potential (2.8 V versus NHE). However, in solar-driven reactions, their role has not been recognized as pivotal as frequently as in UV-excited systems. Photocatalytically, Bi_2_WO_6_ films [23] were shown to generate ^**·**^OH under visible-light illumination. As expected, the films were unable to generate ^**·**^O_2_^–^ under the same conditions, as proven by dimethylsulfoxide (DMSO) ESR trapping experiments (Fig. 4D). This showed that the main effect behind the generation potential of radicals is the position of the valence band (VB) and conduction band (CB) for generation of ^**·**^OH and ^**·**^O_2_^–^, respectively.

### Holes

Photogenerated holes have been shown to play a major role in the degradation pathway mechanism in many reports (by both direct oxidation and the formation of reactive radicals) [17, 24, 25]. In aqueous experiments, they are most commonly scavenged by ethylenediamine tetraacetic acid (EDTA) or ammonium oxalate, but other researchers have used also oxidation of terephthalic acid to its hydroxy form, which has been shown to proceed via direct oxidation by holes [26]. Recently, a visible-light-responsive self-assembled perylenetetracarboxylic diimide (PDI)/reduced graphene oxide (rGO) composite film was obtained and showed good photocatalytic performance and efficient interfacial steam generation [27]. Here, the recombination of e^–^–h^+^ pairs was used to produce the photothermal effect of the films by above-bandgap e^–^ and h^+^ of the upper nano-PDI relaxed to the edge of the band and converted the excess energy into heat. The thermal effect accounted for ~11% of the total degradation.

Overall, the radical species that are responsible for the degradation of organic pollutants do not differ when using films versus suspended systems. The differences that arise among the studies are more commonly ascribed to the different synthesis methods [20] or the presence of potential *p*–*n* junctions, which greatly affect the photocatalytic activity as discussed below. In the next section, we discuss strategies for increasing the number of radical species, thereby increasing the efficiency of the photocatalytic process.

## Common Strategies for Achieving Solar-Light Activity

Many strategies can be applied to coated photocatalysts to enable their operation under solar irradiation. It is worth noting that the strategies here do not differ substantially from the strategies applied with heterogeneous photocatalysts in powder form. However, for better understanding, we state the strategies briefly as well as corresponding directions for more detailed reviews on specific topics.

### Doping

Doping is the intentional introduction of impurities into the crystal structure of a semiconductor to change its optoelectronic properties. In a recent review paper, Pedanekar [28] provided a comprehensive overview of various chemical and physical deposition methods for the preparation of metal oxides (TiO_2_, ZnO, WO_3_, Bi_2_O_3_, SnO_2_, Cu_2_O, Fe_2_O_3_, and CeO_2_) and sulfides (CdS and ZnS) in thin film form, which are well-known photocatalysts for water treatment. The introduction of new energy levels into the bandgap by doping with metals or nonmetals leads to a red-shift in these materials, making them much more efficient under visible light. Interstitial or substitutional doping of nonmetals into the crystal lattice forms new hybrid orbitals within the bandgap, narrowing it. This mechanism in TiO_2_ is well described in several reviews [29, 30]. Among nonmetals, doping with C and N using a variety of chemical or physical methods seems to be the most promising approach. The two most influential papers are about N-doped TiO_2_ [8] and C-doped TiO_2_ [31], the latter describing a 20-fold increase in the visible-light activity of such films. In addition to forming hybrid orbitals from 2*p* orbitals of N and O, nitrogen doping can also form impurity energy levels above the valence band, which also reduces the bandgap [8]. Doping with metals (V, Cr, Ni, Mn, Fe, Au, Pt, and Ag) is much more efficient at trapping free electrons and prevents electron–hole recombination. For example, the presence of Fe creates additional Fe^3+^/Fe^2+^ and Fe^4+^/Fe^3+^ levels in the CeO_2_ band structure, reducing the bandgap energy [32].

Sulfur also effectively narrows the bandgap of TiO_2_. It can be doped as an anion to replace oxygen or as a cation to replace titanium [33], while N/S codoping has a synergistic effect on the photocatalytic efficiency under visible-light illumination. The source of dopants and their content are also important, as a decrease in efficiency has been observed when adding excessive amounts of dopant [31, 32].

Lately, new type of materials—hybrid organotitanias—have emerged [34, 35]. They are obtained using in situ incorporation of dyes, nonmetals, or organic compounds such as phenylendiamine or tyrosine during the synthesis of TiO_2_. The method allows fine-tuning of the crystalline structure, size, and shape by simply varying the pH of the synthesis gel. The incorporation of such moieties (crystal disruptors) into the crystal structure of titania enables these materials to absorb light in the visible range owing to both a decrease in their bandgap and the presence of additional absorption edges at wavelengths longer than 400 nm.

### Surface Grafting

**Fig. 5:**
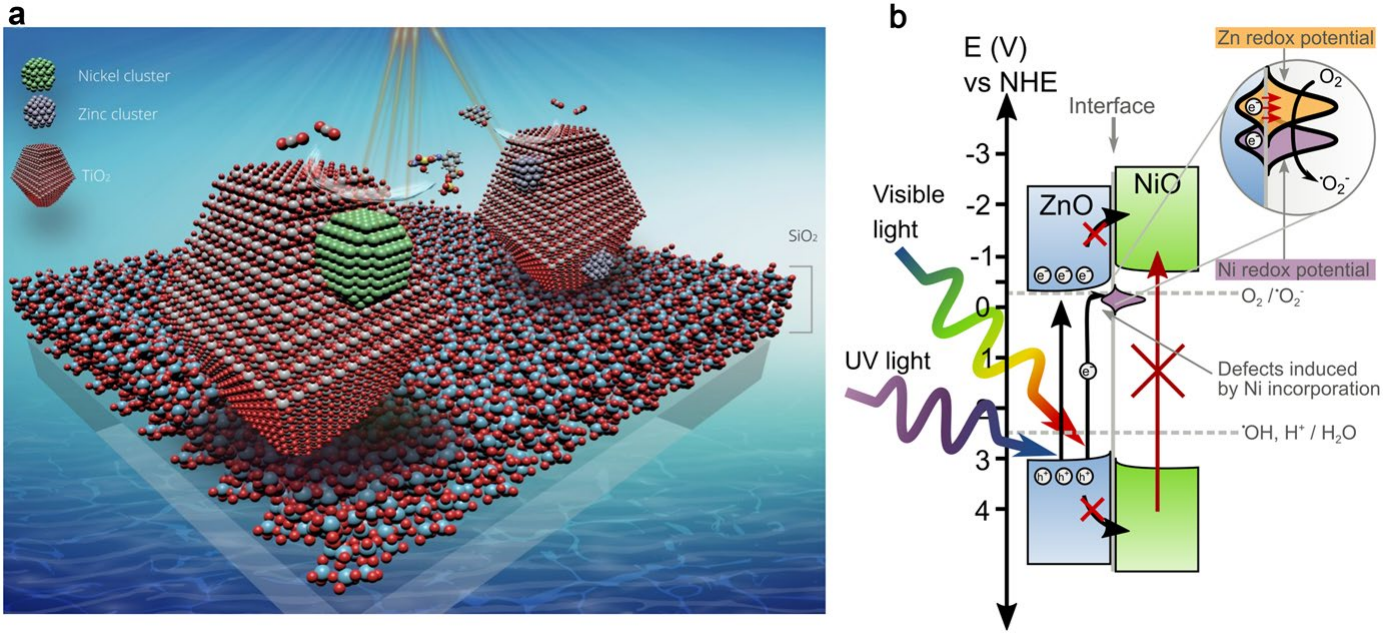
Schematic representation of IFCT mechanism of Ni and Zn clusters on TiO_2_ NPs immobilized on glass slides (**a**) [17]. Potential diagram of ZnO loaded with Ni oxo clusters (**b**). Potential co-grafting with Zn clusters is also represented in the inset

Surface grafting describes the creation of clusters of very small nanoparticles on the surface of a photocatalyst. These are usually in the form of oxo-clusters or metal nanoparticles. Excellent reviews on the subject of surface modification have been written by several authors [36, 37]. Herein, we limit the term “grafting” to the sense of chemical groups loaded on the surface, such as carboxyls, carbonyls, and hydroxyls. Quantum dot (QD) sensitization could also be considered here. Sun et al. prepared ordered TiO_2_ nanotube arrays where CdS QDs were deposited into the pores of the nanotube arrays by a sequential chemical bath deposition method [38]. The generated photocurrent response of the CdS-modified electrodes was 35 times higher than that of a plain TiO_2_ nanotube film electrode. The cell efficiency was 4.15% for the as-modified CdS–TiO_2_ nanotube film. In this section, we focus on stacking the surface of a photocatalyst with metal or metal-oxo clusters. On the surface, these typically exhibit a high degree of structural elasticity. Combined with the ability to change their oxidation state, as is the case with transition metals, this provides a good source of sites for charge carriers to migrate into [17, 39–42]. Thus, the effectiveness of this approach for solar applications relies on a reduction of the electron–hole recombination as well as the ability to act as co-catalysts (Fig. 5a); i.e., since the clusters are reduced/oxidized on the surface, they can play a role in redox processes on the surface itself with the desired reactants (Fig. 5b). Nakajima et al. [39] have suggested that grafting also enhances the crystallinity of the annealed samples, thereby promoting the mobility of electron holes. However, one of the main mechanisms for this approach is based on so-called interface charge transfer (IFCT), as demonstrated experimentally by an in situ X-ray absorption near-edge spectroscopy (XANES) experiment [43, 44]. Another set of in situ measurements was carried out by Šuligoj et al. using electron paramagnetic spectroscopy [17]. This experiment clearly showed the presence of ^**·**^O_2_^–^ radical as well as superfine structure of Ni cluster on TiO_2_ in 3+ oxidation state, which was upon illumination reduced to Ni^2+^, visible in electron paramagnetic resonance (EPR) spectrum.

As expected, here also research into films and immobilized photocatalysts has mainly focused on grafting the surface of TiO_2_ [17, 41]. However, when metal nanoparticles are grafted onto the surface of a catalyst, plasmonic effects can occur; That is, metal particles exhibit the rather unique property of enhancing the sunlight yield through localized surface plasmon resonance (LSPR). This phenomenon is an optical property that arises from the collective oscillation of free electrons on the surface of metal nanostructures when the wavelength of the incident photons matches the natural frequency of the oscillation of the free electrons of the plasmonic metal surface, causing a strong oscillation of the surface electrons. In other words, the formation of resonant SP states leads to an enhancement of the local electric fields near the metal NPs. When a semiconductor is brought near a grafted metal, it encounters these intense electric fields, leading to the rapid formation of e^–^–h^+^ pairs in the semiconductor. Such phenomena have been exploited successfully in films activated by sunlight by using Cu [45], Au [46–48], and Ag [49–51]. It is worth noting that the electromagnetic near field at the plasmon resonance decreases rapidly, within 100 nm [52] from the plasmonic metal surface, effectively promoting surface reactions rather than generating charge carriers in the bulk of the material. Therefore, careful control of the thickness is important; For example, in photoelectrochemical water splitting using hematite modified by Au-NP at the surface, higher enhancement was observed in thin compared with thicker hematite films, which can be attributed to the shorter charge transport distance and the optimal effect of local field enhancement.

### Semiconductor Coupling

The semiconductor coupling approach differs from grafting mainly in that larger and fully crystalline nanoparticles are used. Such particles have lower structural elasticity but offer higher crystallinity and thus higher stability, as well as better absorption of incident radiation. Coupled semiconductors can be made by combining a semiconductor with another material with a different bandgap width. In this case a heterojunction is formed [53, 54]. This is one of the most widely used strategies for enhancing the solar light applicability of immobilized photocatalysts [55–62]. In particular, the heterojunction of various semiconductors with TiO_2_ is found to be widely used in the analyzed literature [18, 55, 63, 64]. Appropriate candidate semiconductors for the formation of a heterojunction with TiO_2_ are Fe_2_O_3_, CdS, ZnO, Cu_2_O, Bi_2_O_3_, WO_3_, g-C_3_N_4_, etc. [8, 65–68].

**Fig. 6:**
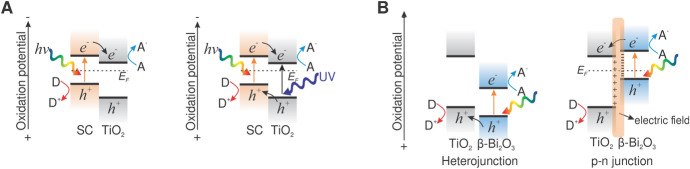
(**A**) Scheme of the transfer of charge carriers under visible-light illumination (left) when TiO_2_ is coupled with a low-bandgap semiconductor (SC), and under UV–Vis illumination (right). (**B**) The formation of the heterojunction (left) and the *p*–*n* junction (right) between TiO_2_ and β-Bi_2_O_3_ and plausible charge carrier migration cascade under visible-light illumination

With such modification, enhanced separation and longer lifetime of the charge carriers is achieved, which improves charge transfer to the adsorbed species [69]. Two photocatalytic activation mechanisms occur in coupled semiconductors: (1) illumination of one semiconductor (the one with the lower bandgap), and (2) illumination of both semiconductors simultaneously. In the first case, the configuration would allow, on the one hand, the absorption of radiation in the visible region of the light spectrum—since the low-bandgap semiconductor acts as a sensitizer for visible light—and, on the other hand, to retard electron–hole recombination in the low-bandgap semiconductor by allowing TiO_2_ to act as a sink for charge carriers generated by visible light (Fig. 6A, left). In the second case, under UV or sunlight irradiation, the semiconductors can conversely act as sinks for the charge carriers generated in both semiconductors, thus extending their lifetime (Fig. 6A, right). When irradiated with UV light, a high concentration of electrons would occur in the conduction band of TiO_2_, since not only the electrons generated by TiO_2_ but also the e^–^ transferred from the conduction band of the low-bandgap semiconductor would accumulate there. In contrast, the VB of the low-bandgap semiconductor contains not only the holes generated by the low-bandgap semiconductor but also the holes transferred from the valence band of TiO_2_. For this purpose, both semiconductors must have different energy levels from their corresponding VB and CB. This strategy improves the spatial separation of the carriers, but at the expense of using carriers with lower oxidation/reduction potentials [70]; i.e., the carriers that accumulate are positioned at lower potentials than when they were photoexcited [71].

Another strategy is called the Z-scheme [70]. Compared with the above-described case, here the photogenerated electrons with strong reducibility remain in the semiconductor with the more negative potential while the holes with the more positive potential remain in the VB of the second semiconductor. Meanwhile, recombination between the charge carriers with lower redox potential takes place at the interface between the two photocatalysts [72, 73].

Yet another way of using semiconductor coupling in films was described by Kwon et al. [2] using the near-IR part of the solar spectrum for injecting additional photons into C-doped TiO_2_. This was done with the use of tridoped β-NaYF_4_:Yb^3+^, Tm^3+^, Gd^3+^ upconversion (UC) nanorods embedded in a C-doped mesostructured TiO_2_ film. The use of the NIR part of the spectrum seems counterintuitive but is enabled by the anti-Stokes emission process of the lanthanide ions.

Focusing on coupling with TiO_2_, tungsten trioxide (WO_3_) is a relatively narrow bandgap (2.6 eV [74]) *n*-type semiconductor which is inexpensive, low in toxicity, and stable in acidic and oxidative conditions and thus an adequate choice to be coupled with TiO_2_. Under UV-light irradiation of TiO_2_–WO_3_ composites, UV-light-generated electrons in TiO_2_’s CB will transfer to that of WO_3_, as the conduction-band edge of WO_3_ is located at a more positive potential than the one of TiO_2_, while photogenerated holes in the WO_3_ valence band will transfer to TiO_2_ [74, 75]. The result is that the recombination rate of the photogenerated charge carriers can be decreased and consequently the photocatalytic efficiency/activity of TiO_2_–WO_3_ composite is improved in comparison with pure semiconductors [76–79]. Patrocinio et al. [80] fabricated TiO_2_/WO_3_ films using the layer-by-layer (LbL) technique followed by heat treatment and used them as self-cleaning photocatalytic surfaces. The LbL technique is a promising, highly scalable method based on the self-assembly of films on a substrate due to attractive (generally electrostatic) forces between different species, allowing the fabrication of uniform thin films with well-defined morphology, composition, and thickness. The prepared LbL TiO_2_/WO_3_ films exhibited higher acetaldehyde degradation rates than pure self-assembled TiO_2_ films. The heating post-treatment of the LbL TiO_2_/WO_3_ films enabled efficient interconnection between the TiO_2_ and WO_3_ nanoparticles and the formation of heterojunctions, which were crucial for the transfer of the charge carriers generated by UV light. The migration of charge carriers between the semiconductors decreased the recombination rate of the charge carriers and extended their lifetime compared with the charge carriers in the LbL TiO_2_ films and consequently increased the photocatalytic activity of the LbL TiO_2_/WO_3_ films for the degradation of acetaldehyde under UV-light irradiation. Soares et al. [81] used a spin-coating technique to obtain thin films of TiO_2_ and TiO_2_/WO_3_ fibers on glass substrates previously prepared by electrospinning. The prepared films were used for UV-light-triggered photocatalytic degradation of Methyl Orange (MO) dissolved in water. The MO degradation results also confirmed that the coupling of TiO_2_ and WO_3_ enhances the photocatalytic activity of the films by: (1) decreasing the bandgap and (2) inhibiting charge carrier recombination as the charge carriers generated by UV light are transferred between the oxides.

Another appropriate candidate to form a heterojunction with TiO_2_ is bismuth(III) oxide (Bi_2_O_3_). Four Bi_2_O_3_ polymorphs are mostly used and investigated: face-centered cubic (δ-Bi_2_O_3_), tetragonal (β-Bi_2_O_3_), body-centered cubic (γ-Bi_2_O_3_), and monoclinic (α-Bi_2_O_3_). The bandgap energy of Bi_2_O_3_, depending on which Bi_2_O_3_ polymorph is used, ranges between 2.4 and 2.8 eV. β-Bi_2_O_3_ exhibits the lowest bandgap energy of 2.4 eV, notably lower than that of α-Bi_2_O_3_ at 2.8 eV. This means that β-Bi_2_O_3_ can absorb visible light across a wider region and therefore appears to be the most adequate candidate for all Bi_2_O_3_ polymorphs to form a heterojunction with TiO_2_ and thus boost the visible-light photocatalytic activity of TiO_2_-based catalysts. There are three unfavorable properties of β-Bi_2_O_3_ which are the reason why pure β-Bi_2_O_3_ exhibits very low photocatalytic activity in heterogeneous photocatalysis. First, the synthesis procedures of pure β-Bi_2_O_3_ generally produces materials with very large particles and consequently low specific surface (below 1 m^2^/g) [82]. Second, the narrow bandgap of β-Bi_2_O_3_ favors recombination of the generated charge carriers [82, 83]. Third, fast charge carrier recombination occurs, as the CB potential of β-Bi_2_O_3_ is too low to oxidize surface O_2_ to ^**·**^O_2_^−^ through a fast single-electron reaction [84]. Figure 6B clearly shows the different roles of each component in such TiO_2_ + β-Bi_2_O_3_ composites. The *p*-type semiconductor β-Bi_2_O_3_ acts as a visible-light photosensitizer and the TiO_2_ as a sink for visible-light-generated β-Bi_2_O_3_ charge carriers, as the bandgap energy of TiO_2_ is too high to trigger its photocatalytic activity under visible-light illumination. In TiO_2_ + β-Bi_2_O_3_ composites, visible-light-generated holes in the β-Bi_2_O_3_ valence band can transfer to the upper lying valence band of TiO_2_ via a heterojunction (Fig. 6B, left) [85]. If there is tight chemical bonding between the *p*-type semiconductor β-Bi_2_O_3_ and the *n*-type semiconductor TiO_2_, a *p*–*n* junction can be established between them (Fig. 6B, right). This means that an equilibrium state of the semiconductors’ Fermi levels (*E*_F_) is established after a *p*–*n* junction is formed as the Fermi level of TiO_2_ is moved down while the Fermi level of β-Bi_2_O_3_ is moved up. This in turn results in the formation of an inner electric field at the surfaces of the semiconductors [86]. The formation of a *p*–*n* junction between TiO_2_ and β-Bi_2_O_3_ enables the feasible transfer of visible-light-generated electrons in the conduction band of β-Bi_2_O_3_ to the CB of TiO_2_. Zou et al. [87] designed a novel photocatalytic reactor with a Bi_2_O_3_/TiO_2_ photocatalyst film coated on hollow glass balls by the sol–gel method to degrade MO under visible-light illumination. The thickness of the Bi_2_O_3_/TiO_2_ film was around 210 nm, and the hollow glass balls were able to float on the surface of the MO solution. A plausible photocatalytic reaction mechanism under visible-light illumination, proposed by Zou et al. [87], shows that illumination with visible-light generated holes in the Bi_2_O_3_ valence band (1.30 eV) and consequently electrons in the CB of Bi_2_O_3_ (−1.41 eV). Electrons in the CB of Bi_2_O_3_ reacted with O_2_ and formed ^**·**^O_2_^−^ radicals. The heterojunction at the border between the Bi_2_O_3_ and TiO_2_ enabled the transfer of the electrons to the CB of TiO_2_ and thus inhibited e^–^–h^+^ recombination. At the same time, holes in the VB of TiO_2_ reacted with H_2_O and formed ^**·**^OH. The generated ROS were further used to degrade the MO dissolved in the water. The strong interaction between the Bi_2_O_3_/TiO_2_ film and the glass beads was demonstrated by recycling tests, in which the photocatalytic activity of the film remained stable after six repetitions, although it was difficult to prevent friction between the glass balls. Xu et al. [88] deposited α-Bi_2_O_3_–TiO_2_ films onto a glass substrate by using a modified sol–gel method at low temperature (< 100 °C) and ambient pressure. The photocatalytic activity of the prepared films was evaluated by degradation of water-dissolved azo dyes X-3B under artificial solar illumination. The enhanced photocatalytic activity of the α-Bi_2_O_3_-TiO_2_ films in comparison with pure TiO_2_ and α-Bi_2_O_3_ films was attributed to the special film structure, increased OH group density, and narrow bandgap of α-Bi_2_O_3_. Two pathways for the charge carrier excitation/separation process under artificial solar illumination were introduced by the authors. One pathway involves the X-3B dye sensitization process, which includes the excitation of dye molecules by absorbing visible-light photons and subsequent electron injection from the excited state of the dye to the CB of TiO_2_ and α-Bi_2_O_3_. The cation radical that forms on the surface of the catalysts then rapidly undergoes a degradation reaction. The other pathway is a photocatalytic process, which is enabled by the ability of α-Bi_2_O_3_ to generate charge carriers under visible-light illumination. The formed α-Bi_2_O_3_-TiO_2_ heterojunction promotes the transfer of holes generated under visible light in the α-Bi_2_O_3_ valence band to the top VB of TiO_2_. As a result, more holes generated by visible light are captured to trigger the photocatalytic reactions than is the case for single-oxide films.

In recent years, graphitic carbon nitrate (g-C_3_N_4_), containing only Earth-abundant elements such as carbon and nitrogen, has attracted a lot of interest from the scientific community for use as a green catalyst as an alternative to already established catalysts in various applications [89–91]. Pure g-C_3_N_4_ has relatively good chemical stability and a bandgap value that enables it to be photocatalytically active also under visible-light illumination [92]. On the other hand, the applicability of g-C_3_N_4_ as a photocatalyst is limited owing to its low specific surface area and fast charge carrier recombination [1]. Roškarič et al. [93] showed that simple mortar milling of previously prepared g-C_3_N_4_ and TiO_2_ can form a junction between the phases that enables the injection of visible-light-generated electrons from the conduction band of g-C_3_N_4_ to the conduction band of TiO_2_, thus prolonging the lifetime of charge carriers. The charge carrier recombination rate of TiO_2_ + g-C_3_N_4_ was further reduced by annealing of the TiO_2_ + g-C_3_N_4_ composite at 350 °C, which owing to the relative thermal (in)stability of g-C_3_N_4_, increased the interface area between the components and thus enabled better injection of the visible-light-generated g-C_3_N_4_ electrons. Wei et al. [55] fabricated a TiO_2_ + g-C_3_N_4_ thin-film electrode via a dip-coating method using a methanol solution of previously prepared TiO_2_ + g-C_3_N_4_ surface hybrid compounds. The surface hybrid heterojunction formed between TiO_2_ and g-C_3_N_4_ promoted the migration of the photogenerated electrons and holes and greatly improved the phenol degradation and mineralization efficiency in water under simulated solar light and an electric field. Zhao et al. [94] used common glass as the substrate and a simple sol–gel and spin coating process for the fabrication of solid-state Z-scheme TiO_2_ + g-C_3_N_4_ thin-film photocatalysts that showed good photodegradation performance for the treatment of colored dye Rhodamine B and colorless tetracycline hydrochloride under visible-light illumination. Further, they found that the photocatalytic activity of the TiO_2_ + g-C_3_N_4_ thin films was strongly influenced by the mass ratio of g-C_3_N_4_ to TiO_2_ and the number of coating layers.

This brief overview of coupled-semiconductor thin films shows that there are different ways in which the charge carriers can be generated and transferred between semiconductors, depending on which semiconductors are used and the nature of the junction between them. Nevertheless, the result is always the same. The photocatalytic activity of the coupled semiconductor films is improved compared with the photocatalytic activity of noncoupled semiconductor films because the “lifetime” of the generated charge carriers is extended by the transfer of the generated charge carriers, which is made possible by the junction between the coupled semiconductors.

### Defect Engineering

Defects in a crystal structure result in slight nonstoichiometry of the material. In nonstoichiometric compounds, a small fraction of atoms are missing or extra atoms are packed into the ideal lattice matrix. This variation in the composition of solid materials usually leads to significant changes in their optical, chemical, electrical, and magnetic properties, which give nonstoichiometric compounds a wide range of applications in catalysis, ionic conduction, superconductivity, and other fields [95].

The concentration of defects in films can be controlled conveniently by adjusting the annealing temperature and/or time and has been found to be positively correlated with their solar photocatalytic performance and inversely correlated to grain size in 10-nm-thick ZnO films [96]. A direct correlation between the oxygen deficiency in ZnO and bandgap was observed, so thermal treatment under an inert atmosphere was suggested [97]. Similar behavior was observed for titanium dioxide. The oxygen gaps are located between 0.75 and 1.18 eV below the conduction band and act as electron traps. Absorption of visible light allows electrons trapped in these discrete states to move into the conduction band and begin degrading pollutants [98]. However, surface defects can act also as carrier recombination centers [99]. In such cases, the concentration of defects in films was purposely lowered, again, by annealing. In defect-induced TiO_2_ (*m*-TiO_2_), the presence of Ti^3+^ states and defects such as oxygen vacancies (O_v_) forms midgap trapping states near to the CB minimum and VB maximum, respectively, which allows much favorable transitions due to the narrower energy level, leading to a narrowing of the bandgap of *m*-TiO_2_. However, as these states are located at a lower (Ti^3+^) or higher (O_v_) redox potential, this inevitably lowers the redox potential of the generated charge carriers. In many cases, the trade-off is still positive, that is, in favor of inducing the defects, yet researchers should be aware of this fact.

**Fig. 7:**
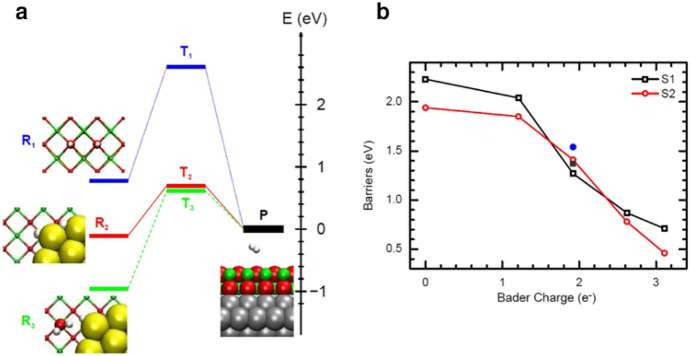
Schematic reaction energy profiles (**a**) for H_2_ generation on MgO(2ML)/Ag(001) without/with the gold cluster. **b** Barriers for water dissociation as a function of inserted excess electrons of the gold cluster controlled by several inserted defects for water molecules on S1 (squares) and S2 (circles) sites. Reprinted with permission from the American Physical Society [100]

Ding et al. [100] showed that, in a perfect MgO(100) substrate without defects grafted with gold clusters, the quantum well state QW1 is the highest occupied molecular orbital (HOMO) of the gold cluster while QW2 is the lowest unoccupied molecular orbital (LUMO) (Fig. 7a). Because OH binds strongly to the gold cluster and cannot diffuse away, efficient water splitting cannot be achieved. By inserting one to four Al_sub_ defects randomly into the substrate, the excess electrons of the Au cluster increase from 0 to 3.11e. More strikingly, *E*_a_ drops from 2.23 to 0.46 eV (Fig. 7b). This way, the barrier for water dissociation with an O_vac_ defect, which is 1.37 eV on the S1 site and 1.54 eV on the S2 site, is significantly reduced compared with the barrier on the defect-free MgO substrate.

In summary, the strategies applied for boosting the solar-driven photocatalytic activity of coatings are not different from those used for photocatalysts in powder form. Therefore, the transition from suspended reactors to immobilized systems should not be delayed by the lack of modification options. There ought to be other reasons for reaching only a 15% share of the immobilized system. Tentatively, more complicated synthesis methods as well as issues in the characterization techniques of films have held back researchers in their usage of coatings. We now focus on the former, that is, the synthesis methods used to prepare films and coatings on various substrates.

## Synthesis Aspects of Films

An analysis of the literature (Fig. 1E) shows that the most widely applied methods for producing coatings are spin and dip coating, plus chemical bath deposition (CBD). All the methods have in common their ease of use and wide availability in laboratories. However, synthesis can be separated into films where semiconductor NPs are formed before coating and those where NPs are formed during coating or during film curing. Herein, we provide a brief overview of the mentioned methods, with an emphasis on the most widely used applications according to the coating method.

### Spin Coating

The advantage of spin coating is the easy control of the thickness and its low consumption of reagents. It also offers the use of a static or dynamic technique; in the latter, the drop is dropped onto the sample at its full rotational speed rather than onto a static substrate [101]. It produces highly uniform layers with good adhesion properties. On the other hand, the substrate size is limited, and more importantly, their shape must conform to a planar geometry. Spin coating is used mostly in applications for degradation of dyes (share 39%), water splitting (share 15%), and degradation of contaminants of emerging concern (CEC, share 12%).

### Dip Coating

Dip coating is a well-established technique characterized by controlled dipping and withdrawal speeds that control the thickness of the applied coating. It is widely used in industry and offers several advantages, such as the ability to coat almost all shapes and sizes, simple design, and controllable parameters (humidity, dipping/withdrawal speeds, temperature, etc.). It suffers from high consumption of reagents and thus large waste generation. It is commonly used in applications such as degradation of dyes (share 35%) and CECs (share 29%).

### Chemical Bath Deposition

Chemical bath deposition (CBD) encompasses a variety of routes for producing functional semiconductor films at relatively low temperature (< 100 °C), requiring only that a substrate be placed in a vessel containing a supersaturated solution of dilute aqueous precursors such as metal salts, complexing agents, and pH buffers. Films of dozens of single- and multi-component oxide materials have been synthesized, mostly from aqueous precursor solutions, on substrates that vary widely in their chemistry and topography, for example, glass rings [102] or glass tubes [103]. We include in this approach also hydrothermal growth on a solid carrier, sometimes called hydrothermal-assisted CBD, which is especially used in photoelectrochemical (PEC) applications [104, 105]. CBD is mostly used for degradation of dyes (share 35%), hydrogen evolution (share 20%), and CO_2_ reduction (share 12%).

## Parameters of Films Affecting Their Solar Photocatalytic Activity

As mentioned above, the reasons for using coatings cannot just be simplicity of application or environmental friendliness, since no separation after the reaction is required. We go into a little more detail about these advantages here. In this section, we discuss some of the key parameters that affect their activity under sunlight. The assumption here is that the phase composition of the films remains the same, including the crystallinity, which in effect means that these parameters are excluded from affecting the activity [106], as they were described in previous sections.

Because photocatalysis includes a series of surface reactions, it may be detrimental to always have three-dimensional (3D)-accessible particles present in the reaction system at all times. Restricting the active surface to a two-dimensional (2D) system can be beneficial owing to the possibility of better control over the reduction and oxidation parts of the film. This is related to the direction of the incident light, which can be controlled and used to guide the chemical reactions, a feature that is only accessible in immobilized systems owing to the static nature of films; For example, Pang et al. used Pt–BiVO_4_/WO_3_/FTO photocatalyst sheets to produce hypobromous acid (HBrO) by oxidation of bromide (Br^−^) under simulated solar light [107]. They observed that the rate of formation of HBrO decreased quickly with the photoirradiation time. This decrease was due to the formation of H_2_O_2_ during the photocatalytic oxidation of Br^−^ and suggested that the strong e^–^/h^+^ recombination on the surface of powder photocatalysts as well as unwanted side reactions that occur in the reaction solution (decomposition of HBrO by H_2_O_2_ produced from the reduction of O_2_ on Pt cocatalyst) were the causes of this phenomenon. The authors managed to produce BiVO_4_/WO_3_ photocatalyst immobilized on a glass slide with a Pt coating that produced more than 80 μM of HBrO (12.9 ppm of free bromine), almost eight times more than the amount of HBrO produced over the Pt/WO_3_ powder photocatalysts. The HBrO concentration in the resulting solution was sufficient for effective disinfection of most microorganisms, such as *Escherichia coli* (0.15–4 ppm), *Pseudomonas aeruginosa* (0.2–1.5 ppm), polio virus (1.9–9.5 μM), and *Cryptosporidium parvum* (5 ppm) in drinking water, swimming pools, and meat processes.

**Fig. 8:**
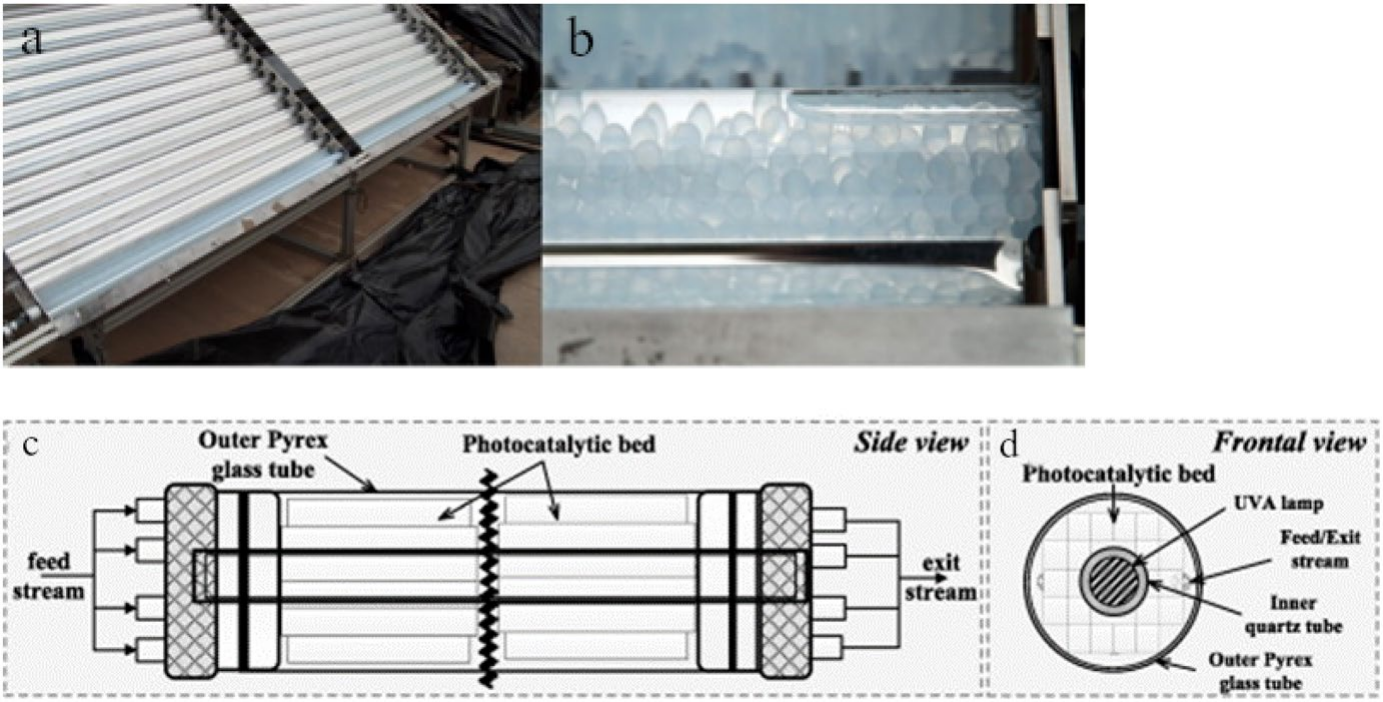
CPCs prepared for tests with immobilized TiO_2_ (**a**) and detail of one absorber tube filled with TiO_2_-coated spheres (**b**) [110]. Schematic representation of CPC pilot unit using cellulose acetate monolith-supported TiO_2_: side view (**c**) and front view (**d**). Reprinted with permission from Elsevier [113]

Moreover, for pilot- or industrial-scale photocatalytic applications, the application of coatings is a benevolent way of increasing the applicability of the process. This has been seen in many studies applying photocatalytic coatings at pilot-scale solar reactors (Fig. 8) [108–113]. The corpus of published literature reported herein reveals that the share of immobilized photocatalysts in pilot studies is 23%, which is 8 percent points higher than in the general corpus of solar-active photocatalytic materials (15%, Fig. 1D).

**Fig. 9:**
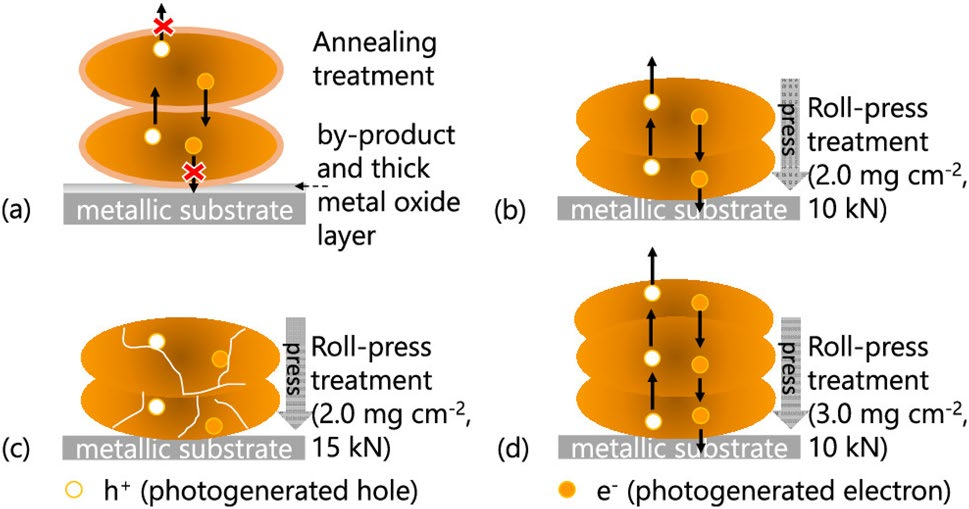
Schemes showing the effects of film structure, compaction density, and thickness on e^–^ and h^+^ transport for a Ta_3_N_5_/Ti electrode fabricated under different conditions: **a** annealing treatment at 773 K under NH_3_ atmosphere and **b**–**d** roll-press treatment under **b** 10 kN with loading per area of 2 mg cm^–2^, **c** 15 kN with loading per area of 2.0 mg cm^–2^, and **d** 10 kN with loading per area of 3.0 mg cm^–2^. Reprinted with permission from the American Chemical Society [114]

Several factors affect the performance of films under solar radiation. Two of these are obviously the thickness and the structure of the film. Subsequently, Xiao et al. added the densification degree to the lust of main factors affecting their performance (Fig. 9) [114]. They were able to study the effect of thickness and compression density by means of roll-press fabrication of Ta_3_N_5_ photoelectrodes on Ti metal sheets. The two factors strongly influence the electron–hole dynamics in the films. Applying too much compression force may result in crushing of the NPs, thus generating new particle boundaries that create locations for e^–^–h^+^ recombination, while excess particle loading (commonly expressed in mg cm^−2^) results in shading of lower-lying NPs and increases the charge carriers’ diffusion path length.

### Effect of Photocatalyst Phase

The structure or phase of the semiconductor or semiconductor system is a key parameter defining its activity. There are countless reports on tuning the crystallinity to boost the activity [35, 115].

While appropriate crystallinity is of paramount importance for high catalytic activity, it has been suggested that the presence of a small amount of an amorphous phase may be beneficial also [116]. For visible-light applications, this is important since, concerning titania, the energy bandgap of amorphous TiO_2_ is 3.0 eV, i.e., 0.2 eV lower than that of anatase [116].

In nitrogen fluorine-doped titania (NF-TiO_2_), the presence of brookite/anatase heterojunctions was found to be beneficial owing to its photocatalytic activity towards microcystin LR degradation under both visible and UV irradiation [117]. The boost in activity is most probably a consequence of rapid interparticle e^–^ transfer between the constituent titania nanocrystals that slows down recombination losses, similar to the highly active mixed-phase (rutile/anatase) titania photocatalyst Degussa P25 [117]. Combining different phases has a profound effect on the activity of films, and more detail on this aspect can be found in Sect. 3.3.

A substantial breakthrough in PEC activity was the construction of multilayer electron donor–acceptor thin films or sensitized colloids in which individual nanosheets mediate light-driven electron transfer reactions [118]. In this case, when sensitizer molecules were “wired” to IrO_2_·*n*H_2_O nanoparticles, a dye-sensitized TiO_2_ electrode became the photoanode of a water-splitting photoelectrochemical cell. In TiO_2_ dye-sensitized solar cells, to enhance the light harvesting capacity, high dye loadings are desirable. Also here, a multilayer approach has proven successful; For example, compared with Eosin Y–TiO_2_ and monolayer-Eosin Y–Fe^3+^–TiO_2_, multilayer-Eosin Y–Fe^3+^(1:1)–TiO_2_ showed higher light harvesting efficiency and visible-light photoactivity for H_2_ evolution [119]. Fe^3+^ linked not only TiO_2_ and Eosin Y but also different dye molecules to form a multilayer dye structure via chemical bonding, thus enhancing light harvesting efficiency and overcoming the quenching and insulating effect of the dye layers. Multilayer N-doped graphene photoelectrodes have even been prepared to work as both semitransparent electrodes and efficient photoelectrocatalysts for H_2_ evolution at low bias [120], thus avoiding the need for expensive substrates such as fluorine-doped tin oxide (FTO), indium-doped tin oxide (ITO), or aluminum-doped zinc oxide (AZO) and relying on omnipresent C as the sole source for the substrate and active material.

In the next sections on parameters, it is assumed that the crystallinity is the same and thus the photocatalytic system behaves the same, although this is not always the case. Assuming this, we discuss the next important factors.

### Influence of Film Thickness

Thickness is one of the crucial parameters affecting the performance of solar-driven photocatalytic films. It is therefore surprising that this parameter is not only seldomly varied and optimized in studies but also rather infrequently reported. Indeed, a thorough review of 110 papers that reported the deposition method for producing solar-active photocatalytic coatings revealed that 35% of them lacked information on film thickness. This percentage is high, and the authors would prefer to see a better description of this parameter across literature. Here, we consider the thickness of the active layer or, in the case of multilayered films (see above), the combined thickness of the active coatings.

As an example, the optimal thickness of TiO_2_ films fabricated by ultrasonic spray pyrolysis for photocatalytic self-cleaning applications was in the range of 170–230 nm [121]. The optimum thickness was supposed to be induced by the reflectance-assisted absorbance, supported by the borosilicate glass/TiO_2_ interface, which could promote additional generation of charge carriers. This effect was confirmed also by Mohan et al. [122], where lower optimal thicknesses were needed in case of Al and Cu support than glass due to the lower absorptivity of the bandgap photons (the photocatalyst was In_2_O_3–*x*_(OH)_*y*_), thus increasing the chance of reabsorption of the reflected photons.

The optimal thickness of WO_3_ films prepared by anodizing W foils was found to be 1 μm [123], while for Bi_2_WO_6_ films on stainless-steel mesh [124], the optimal thickness for 4-nitrophenol degradation was 627 nm. These optima were ascribed to the large external surface areas and lowered bandgap with films of this thickness. Increasing the thickness eventually increases the film resistance, prolongs the electron transport paths, and thus increases e^–^/h^+^ recombination. It also decreases the transmittance of films, thus reducing the incident light intensity on active species. This is especially true in dye-sensitized solar cells (DSSC). Kao et al. found the optimal thickness for TiO_2_ DSSCs to be 1.5 μm [125]. Similarly, for three-dimensional nanoporous WO_3_ films, the optimal thickness for visible-light degradation of Congo Red (CR) dye was ~ 1 µm [123]. In this case, the authors ascribed this to the large surface area and narrower bandgap.

On the other hand, for much thinner films (10–50 nm), enhanced solar photocatalytic discoloration of Rhodamine B (RhB) dye was found with thinner films (10 nm) of iron phthalocyanine (FePc) deposited on indium tin oxide (ITO) [126]. This was explained by effective e^–^ injection into the ITO substrate for the thinner as opposed to the thicker ones. The injection was facilitated by the large difference between the ITO work function and the FePc LUMO, which highlights the importance of choosing a proper substrate for coatings.

Increasing the film thickness also increases the probability of film flaking. Hence, the optimal thickness must be described not only in terms of a single use but also regarding the long-term stability of the coatings [127]. Most importantly, also here, the goal is to produce as many charge carriers as possible, with the longest possible lifetime. Hu et al. found the optimal thickness of TiO_2_ film supported by vertically ordered Au nanorods superlattice array (O-AuNRs/TiO_2_) to be 85 nm [128]. Thicker films decreased the H_2_ yield slightly due to the fact that the electromagnetic near field at the plasmon resonance only extends up to ~ 100 nm from the plasmonic metal surface.

Diffusion of reactants is another aspect to consider. In general, in solid–gas reactions, a thin catalyst layer is beneficial for improving the contact between the catalyst and gas, while for a transparent, minimally reflective substrate such as glass, a thicker film exhibits improved light utilization due to enhanced photon absorption [122].

It is advised that optimization of thickness be conducted for each individual study for the reasons described above.

### Effect of Film Morphology

The immobilization of a photocatalyst onto a surface results in layers with thickness from a few nanometers to a few micrometers. This can significantly increase the distance for charge carriers to travel and thus the likelihood of recombination events. Therefore, researchers have addressed this problem by changing the coating morphology.

Generally, nanostructuring of semiconductor coatings means moving from single- or polycrystalline materials that exhibit a classic, planar semiconductor–liquid–gas interface to nanoscale or even microscale geometries, i.e., nanoparticles, nanorods, nanowires, nanocones, nanoflowers, etc., that provide a three-dimensional interface with a large surface area. While most of the semiconductor material remains far from the interface in the planar configuration, the open structure and high surface-to-volume ratio of nanostructured films allow the medium to penetrate much of the film. This has dramatic implications for not only the active surface area of the films but also the dynamics of the photogenerated charge carriers (i.e., charge separation and recombination) [129]. This has implications also on a macroscale; For example, Kargar et al. [130] observed large improvements in the photocathodic current when using (3D) ZnO/CuO heterojunction branched nanowires (b-NWs) grown on a copper mesh compared with those grown on copper film owing to the improved surface area caused by the mesh substrate.

Particle size has a direct effect on surface morphology. Imao et al. [131] observed that nanostructured TiO_2_ films with a narrow distribution of particle size (prepared by using polyethylene glycol (PEG) during the synthesis) increased the amount of adsorbed dyes as compared with those without PEG. Also, the performance of the solar cell increased. Grätzel’s team [132] reported an improved photoresponse of nanocrystalline α-Fe_2_O_3_ films owing to optimized silicon doping and the changed morphology, which was strongly influenced by the Si doping, decreasing the feature size of the nanocrystallites. In a tandem-cell configuration with two series-connected dye-sensitized solar cells, the best-performing Fe_2_O_3_ photoanode would yield a solar-to-chemical conversion efficiency of 2.1%, thus showing the importance of accounting for morphology.

**Fig. 10:**
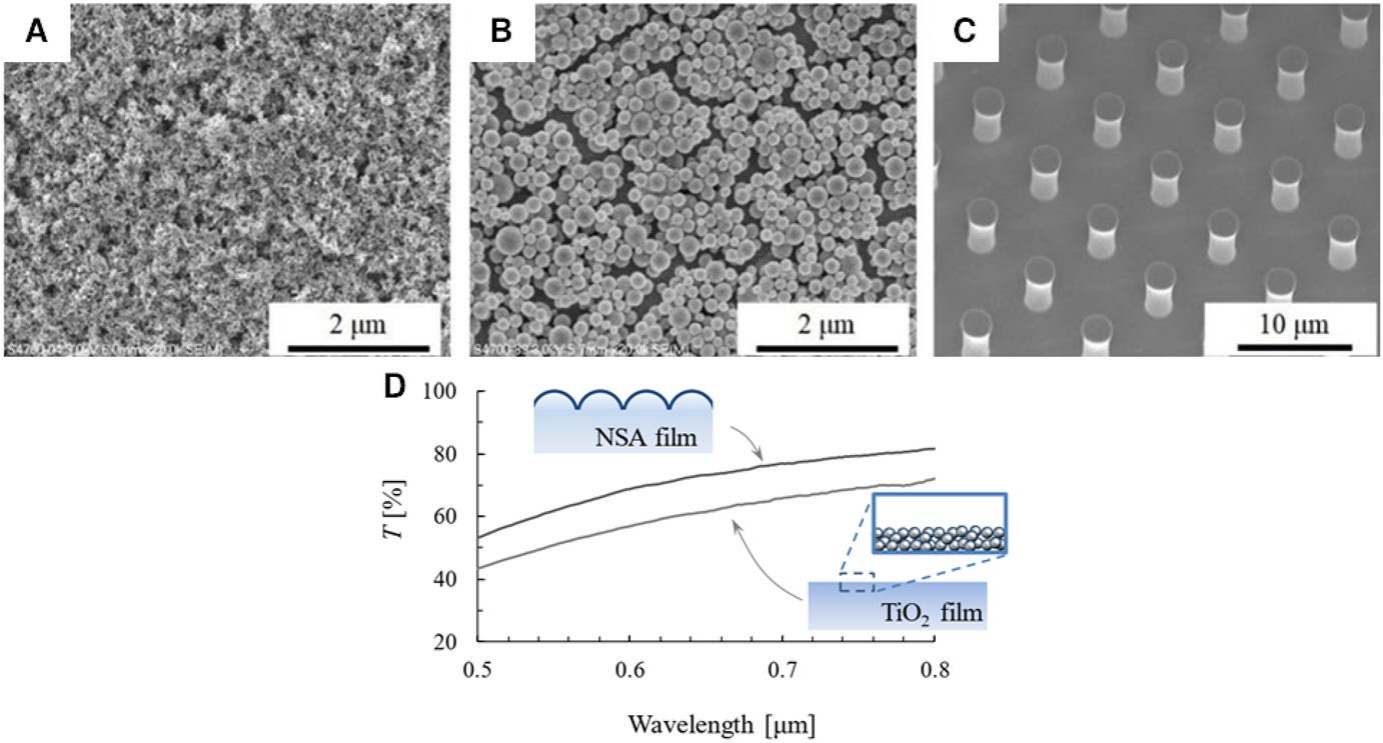
Structures (**A**–**C**) of mesoporous films of TiO_2_. **B** Improved transmittance of spherical nanoparticle aggregate (NSA) film. Reprinted with permission from IOP Publishing [138]

In another example, Hwang et al. observed the characteristics of a carbon film that played a key role in enhancing hydrogen production using ZnO-based photocatalysts [133]. The efficient conduction of e^–^ to the carbon film support led to 64% higher production of H_2_ than in the case of ZnO alone. Films prepared by the doctor-blade method, consisting of BiVO_4_ microflower leaf-like structures, were successfully used to produce 9.52 mmol/h of H_2_ within 7 h from natural lake water [134]. The enhanced photocatalytic performance was attributed to the lower recombination resistance, improved morphology, and good separation and migration of the photogenerated charge carriers. Intense light absorption in the visible region was also reported, which was attributed to multiple scattering of light within the macroporous nanoring structure [135]. This distinct property of the samples was beneficial for enhancing their light utilization ability. The same effect was reported by Song et al. [136], where a highly ordered mesoporous N-doped TiO_2_ inverse opal structure showed excellent light harvesting efficiency and fast mass transport. In general, improved light absorption is a highly common sought-after factor for improving the efficiency through the introduction of more complex morphologies [137]. The size of these structures generally falls in the micron range, except when scattering is to be avoided (e.g., in self-cleaning transparent coatings), and the sizes of such features are preferably smaller than the wavelength of the incoming irradiation (Fig. 10) [138]. Such morphological features are expected to effectively utilize the energy of sunlight in many applications using functional photocatalyst films with optimized design [88].

An interesting approach was developed also by Lee and Lee [139]. A thin TiO_2_ layer spin-coated on a glass substrate was first patterned by embossing and treated with a higher TiCl_4_ concentration than the thicker TiO_2_ layer above. Since the patterned layer was denser and had a higher refractive index than the top layer, it acted as a diffraction grating. Line gratings with a period of up to 1 µm were incorporated into the film. The diffraction efficiency increased with the height of the grating, and efficiencies of over 80% were achieved in the near-UV and visible regions. It was shown that the incorporation of a diffraction grating significantly increases the performance of TiO_2_-based photovoltaic and photocatalytic devices by improving the light output. In one case, WO_3_ nanoplate films on W foil were controlled to contain larger and thinner monoclinic WO_3_ phase by addition of oxalic acid (OA) [140]. Films with such morphology also had a narrower bandgap and higher visible-light absorption. WO_3_ nanoplatelets prepared with OA exhibited enhanced photocatalytic performance for the decomposition of gaseous acetaldehyde under simulated sunlight irradiation, which was ascribed to the enhanced charge separation and light absorption as well as more reactive surface sites.

Additionally, the presence of nanopores as opposed to surface nanotexture features has been shown to be beneficial [123], clearly showing the importance of film morphology. The *E*_g_ value of the nanoporous films was ~ 2.73 eV (~ 460 nm), and despite the quantum confinement of the nanopores, their optical absorbability in the visible-light range was still much better compared with nanotextured films with a bandgap of ~ 3.08 eV.

In this regard, the substrate morphology has also been shown to have an important effect on the photocatalytic performance. Li et al. prepared 90-nm-thick TiO_2_ films on Si wafers with either micro-, nano, or no morphological features (Fig. 10) [115]. The features included nanosized pyramids. The increased photocatalytic activity was attributed to the excellent light trapping properties and enhanced carrier separation with the additional formation of a *p*–*n* junction when doping the Si wafer’s surface with phosphorus.

### Photocatalytic Stability of Films

One of the most important factors for the applicability of photocatalytic materials is their long-term stability, in both the physicochemical and catalytic sense. The former aspect is usually addressed by analyzing spent materials using techniques such as X-ray diffraction analysis [64, 141], scanning electron microscopy (SEM)/transmission electron microscopy (TEM) imaging [142, 143], and there derived energy-dispersive x-ray spectroscopy (EDS) patterns [143], Fourier-transform infrared (FTIR) spectroscopy, atomic force microscopy (AFM) [142, 144], or solid-phase UV–Vis spectroscopy. The overall goal of the aforementioned techniques is to confirm the retention of the overall morphology, crystal structure, and optoelectronic properties. However, in most cases, the number of repeated cycles is low and usually insufficient to detect structural changes. Therefore, the conclusions of such studies are usually positive for confirming the long-term stability of the materials. On the other hand, surface studies focusing on species that block catalytic sites are very scarce. In degradation reactions, these species are usually recalcitrant residues of the parent compounds, while in water oxidation reactions, they may be oxidized species of the parent material, as in the case of Ta_3_N_5_ [145], where prolonged testing led to the formation of an inactive TaO_*x*_ phase. Indeed, for photothermal CO_2_ reduction using In_2_O_3–*x*_(OH)_*y*_ photocatalyst [122], the reduction of the latter by migration of oxygen to the support resulted in an inactive indium metal. Further methods need to be developed for a more complete description of the stability of photoactive films.

## Characterization Methods for Solar-Active Films

Various methods can be applied to characterize the properties of pristine (i.e., unused) solar-active films, depending on which properties are the key factors determining the photocatalytic activity of the investigated solar-driven systems and are therefore the subject of research. Some of these methods were developed to study the surfaces of samples, so no special sample preparation is needed in such cases. Among these methods, X-ray photoelectron spectroscopy (XPS) and AFM are frequently used for thin-film characterization. XPS can be employed to determine the surface electronic states, chemical composition, and purity of the prepared films [87]. AFM is another technique developed to determine the surface roughness of films [146]. SEM, designed for directly studying the surfaces of solid objects, can be used to examine both powder and thin-film samples. When coupled with EDX, it can provide insight into the morphology and elemental composition of the studied films. One of the obstacles is the low electrical conductivity of solar-active films, which can be addressed by the deposition of a very thin (few nanometers) conductive layer on top of the film [146]. The crystalline phases of thick films and their phase content can be determined by XRD analysis in a symmetric geometry, as is most commonly used for bulk samples [147]. In the case of thin films deposited on a glass support, the high background from amorphous silica can be observed in the 2*θ* range from 20° to 40° [148]. A silicon substrate seems a better choice in this case, but owing to its intense reflection at 2*θ* angles above 60°, this angle is the upper measurement limit. The use of a prolonged integration time (500 s per step, for instance) increases the quality of diffractograms obtained from thin films but also extends the duration of such measurements. If only the determination of crystalline phases is required, the range can be narrowed to detect only the most intense diffraction peaks of all the polymorphs [149]. With respect to traditional XRD, grazing-incidence X-ray diffraction (GIXRD) is a more appropriate tool for investigating thin films and surfaces [150]. This technique uses incoming X-ray at small angles of incidence (2*θ* values of 0.5°), the wave penetration is limited, and thus the reflections come only from the surface structure. Very low scattering angles are used in another technique known as grazing-incidence small-angle X-ray scattering (GISAXS), for which a synchrotron radiation source is needed. The setup of such experiments is described in detail in Ref. [151]. The mesostructure, open porosity, and pore architecture of N-doped titania thin films were successfully determined using this method [152]. Micrographs obtained by transmission electron microscopy (TEM) show thin films in plan and cross-section view and complement the information obtained by GISAXS. Sample preparation of Ni-oxide thin films on silicon substrates is described in Ref. [153], and the same procedure can be used also for solar-active films. Teh crystalline structure of the film layers can be characterized also by Raman spectroscopy [87, 146]. The Raman activity of substrates is an issue in thin-film Raman spectroscopy. Very often, the film is too thin (< 100 nm) to avoid light penetrating the substrate, meaning that the Raman spectra of both the film and substrate are detected in the experiment [154]. Gasparov et al. [155] compared a number of thin-film substrates (MgO, LaGaO_3_, LSAT, DyScO_3_, YAlO_3_, LaAlO_3_, NdGaO_3_, SrLaAlO_4_, and SrTiO_3_) to study their applicability in Raman-based thin-film research. According to the results of that work, MgO was found to be the best Raman substrate with the largest free spectrum and low Raman background. LaGaO_3_, LSAT, DyScO_3_, YAlO_3_, and LaAlO_3_ can be considered as good Raman substrates because they show a relatively broad spectral range that is free of phonon modes, while having a flat Raman background of low intensity within this range. The optical properties of films can be analyzed by UV–Vis diffuse reflectance (UV–Vis DR) and/or UV–Vis near-IR (UV–VIS–NIR) spectral measurements [87]. The photoluminescence (PL) emission spectra of the investigated films can be used to investigate the efficiency of charge-carrier trapping, migration, and transfer and to understand the fate of electron–hole pairs in photoactive films [88]. The film thickness can be determined by using a profilometer, weighing the samples (in the case of thick films), or SEM measurements. TO determine the thickness of thin films using SEM, titania thin films were first deposited onto silicon resins. These resins were then positioned perpendicular to the sample holder using a triangular wedge, enabling the observation of the thickness of the thin films. Carbon tape was used to attach the resins to the wedge [149]. On the other hand, a piece of thin film was cut to the size of the holder using a scalpel. After gluing it to the holder with double-sided carbon tape, a few slits were lightly cut on the surface of the film with a scalpel. The film bent in a horizontal direction in some places, allowing the thickness of the film to be evaluated [156].

While these methods are common across the photocatalytic field, some of them that are more specific to immobilized systems are discussed in more detail.

### Adhesion

Adhesion is one of the most important parameters when evaluating the quality of photocatalytic films, along with the film density, stress, grain size, etc. Adhesion can be defined as the molecular attraction that holds a material together (as a single material or in multiple layers). Adhesion can fail via either cohesive failure (breaking up of the main layer) or adhesive failure (breaking at the interface between two phases). In the literature reviewed, researchers generally do not distinguish between the factors responsible for adhesion failure. However, several layers are commonly used, in order to fill the voids in the underlying coatings [17, 134]. This is a clear case of improving the cohesive forces in the film to improve its mechanical stability.

Techniques used for measuring adhesion are applied sparingly in the research community, but the most common are the Wolff–Wilborn test (ISO 15184) [157], ultrasonication [158, 159], the Scotch tape test [160], and the cross-hatch adhesion test according to ASTM D 3359B-02 [161, 162]. The latter seems convenient since the peel strength of 3M 600 tape is 0.44 N mm^−1^. For comparison, a common car with tires on a dry asphalt pavement produces a maximum rolling friction per unit width on each tire of 0.30 N mm^−1^ [163].

Achieving intimate contact between the photoactive layer and the underlying substrate is important also regarding the transport of charge. For instance, in photoelectrochemical (PEC) water splitting reactions, photoexcited electrons migrate from the top of the film to the electrode at the bottom, where they have to jump to the metal collector via electron hopping [164], during which the high-resistance depletion layer and Helmholtz layer are traversed. In many cases, charge carriers experience multiple high-resistance layers connected in series. For example [165], sulfur atoms located at the interface between polymeric carbon nitride (PCN) and FTO acted not only as the initialization for the growth of the PCN films but also as connections to assist charge migration in the PEC reaction.

For the same reasons, also in direct heterogeneous reactions (with no external circuit), achieving intimate contact is crucial. Creating contact between graphene and TiO_2_ thin film led to a large work function difference between TiO_2_ and graphene, which caused e^–^ transfer from graphene to titania [166].

Finally, appropriate adherence is pivotal in most water cleaning reactors, since the flow of liquid exerts force on the layers. Most commonly, adhesion is achieved by means of inorganic binders such as SiO_2_ [17, 167] or SnO_2_ [160] or without binders by using hybrid sol-suspension methods where the sol acts as a binder to the substrate. Huang et al. produced transparent 80-nm-thick films composed of protonated g-CN/TiO_2_ for solar NO abatement [162]. In their case, excellent adhesion was achieved by addition of up to 6.25 wt.% H^+^-rich graphitic carbon nitride (g-CN), where H^+^ could form complexes with metal ions, slowing down the condensation reaction in a more ordered manner and producing crystalline TiO_2_, which could then form bonds with the OH-rich surface of the glass.

### Thermoanalytical Methods

Thermal analysis (TA) describes a group of techniques in which chemical or physical properties of a substance are measured as a function of temperature or time while the substance is subjected to a controlled (heating, cooling, or isothermal) temperature program. Thermogravimetry (TG), dynamic scanning calorimetry (DSC), and coupled TG-MS (mass spectrometry), which allows the determination of the evolved gases with very high sensitivity [168], are the three thermoanalytical methods most frequently used for characterization of photocatalytcally active thin films.

In the available literature, most articles report photocatalytic processes based on TiO_2_ photocatalysis, while a few also report photocatalysis with heterojunctions. Therefore, this subsection also focuses on titanium dioxide. Another point to note is that we focus only on the characterization of films as they appear before the photocatalytic reaction. The aspect of analysis of post-reaction films was discussed in Sect. 5.4. The precursor starting solution contains a precursor salt and various additives, complexing agents, or templates that can be used to tailor the desired properties of the final material. To prevent agglomeration and increase the surface area, various organic polymers can be added to the sol. After the deposition process, the thin films must be treated thermally to ensure proper adhesion to the substrate and obtain a suitable crystalline phase, since untreated films with amorphous structure do not have photocatalytic properties. During the thermal treatment of the initial sample, dehydration, combustion of organic phases, and decomposition of the initial precursor take place in different temperature ranges specific to the particular system, finally leading to the formation of the desired photocatalytically active crystalline phase. With increasing temperature, the crystallites become larger or a phase transformation from anatase to rutile takes place.

The parameters of the thermal treatment (heating rate, final temperature, and duration of the possible isothermal treatment) have a considerable influence on the photocatalytic efficiency of the final material. Besides the crystalline phase, the size of the crystallites and the agglomeration of particles are also important factors [169]. Photocatalytic reactions take place at the phase boundary, thus particles of small size with high specific surface area are desirable, although the bandgap also increases with decreasing particle size. The crystallite size and degree of crystallinity thus have a complex influence on the photocatalytic activity. The recombination rate also depends on the size of the grains. With the help of thermoanalytical techniques, one can determine an appropriate “temperature window” for optimal thermal treatment [156], while the optimal temperature can be determined by using additional diffraction and spectroscopic techniques.

The general approach for using thermoanalytical techniques to determine an appropriate thermal treatment for visible-light photocatalysts is to perform measurements on powder analogs. In Ref. [149], which reports on the high catalytic efficiency of S-, N-, and Pt-doped TiO_2_ thin films (thickness ~ 200–300 nm), TG-DSC-MS measurements were performed to determine the crystallization temperature. The temperature range for anatase crystallization depends on the counterions present in the sol, which also affect the size of the crystallites. The crystallization process becomes possible after counterions are evolved from the structure. For Cr-implanted TiO_2_ films, which are active under visible-light illumination, thermal analysis was again performed for the corresponding xerogel [170]. On the basis of the TG and DSC curves, 400 °C was determined to be the heat treatment temperature, followed by 2 h of isothermal treatment at this temperature. No exothermic peaks were detected in the DSC curve, and the authors concluded that anatase phase was formed during the xerogel preparation, at 75 °C. Another interesting example visible-light photocatalyst is floating fly-ash cenospheres supported AgCl/TiO_2_ films with thickness ~ 2 μm. In this case, the TG-DSC curves showed that the cenospheres decomposed fully at 450 °C, whereas the preparation temperature was 70 °C [171].

Thermal treatment of nanostructured titania films, which are active under UV-light irradiation, was again determined on the basis of xerogel results [172]. The second exothermic peak in the DSC curve, located at 402 °C, was identified as the crystallization temperature of anatase, but TEM micrography revealed that anatase grains with size of 7 nm formed as already at 350 °C. The reason for this discrepancy may lie in the fact that the crystallization process occurs at lower temperatures with regard to the xerogel. The films were treated thermally in the temperature range of 350–500 °C, and the results showed that, with increasing temperature, the size of the nanocrystals increased while the surface area decreased, so higher photocatalytic activity was obtained in the case of less thermally treated films exposed to 350 °C. In a paper reporting on the photocatalytic activity of nanocrystalline titania films prepared by the doctor-blade method from three different pastes, DSC was used to determine the amount of adsorbed water, which correspond to the number of surface hydroxyl groups. DSC was performed on the powdered samples from which pastes were prepared [173].

**Fig. 11:**
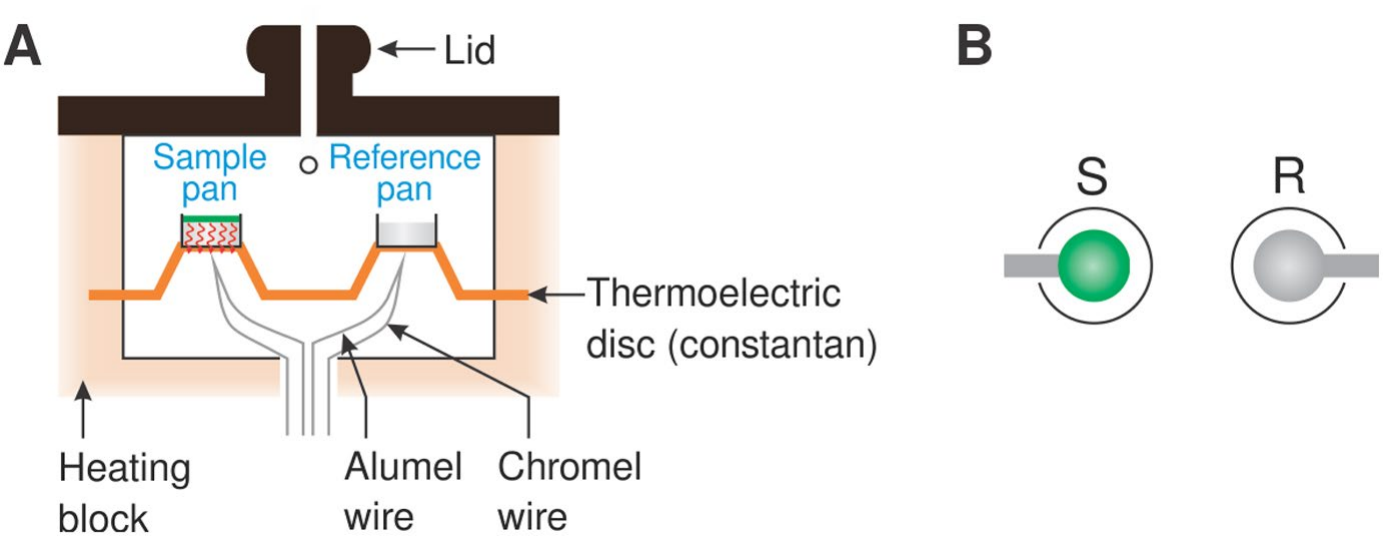
Schematic presentation of DSC measurement of a thin-film sample (green color) deposited on a massive substrate. As the reference, the bare substrate is used. The heat absorbed or evolved from the film is mainly consumed by the substrate itself. **A** A thin film, deposited on a substrate, that can be placed directly on the thermocouple; **B** top view, S—sample, R—reference

Routine analyses are always performed on powder samples, and the results are then applied on film samples. The reason for this strategy is that thermal analysis of thin films is a demanding procedure and direct measurements of thin films are still not very common [174]. The sensitivity of balances in TG instruments is on the order of 1 μg, so detection of the thermal decomposition of thin films is possible [175]. However, the amount of sample available is very small, typically below 1 mg, so the mass change during TG experiments lies in the range of buoyancy and aerodynamic effects. In DSC measurements, the evolved or absorbed heat diffuses into the substrate, thus the measured enthalpies are very small (Fig. 11a) [176]. One possibility is to deposit the thin film on one side of the substrate and place it with the clean side directly onto thermocouples to avoid the additional heat capacity of a crucible (Fig. 11b). The basic strategies for overcoming the mentioned difficulties were published in the 1990s in two review papers [176, 177] and summarized elsewhere [178]. The results obtained for deposited thin-film samples, thin films scratched from the substrates, and corresponding xerogels can differ considerably due to differences in sample size, structure (influenced by substrate), and microstructure [178, 179]. Therefore, it is essential to perform thermal analysis on thin films, deposited on a substrate, to obtain the most reliable results.

**Fig. 12:**
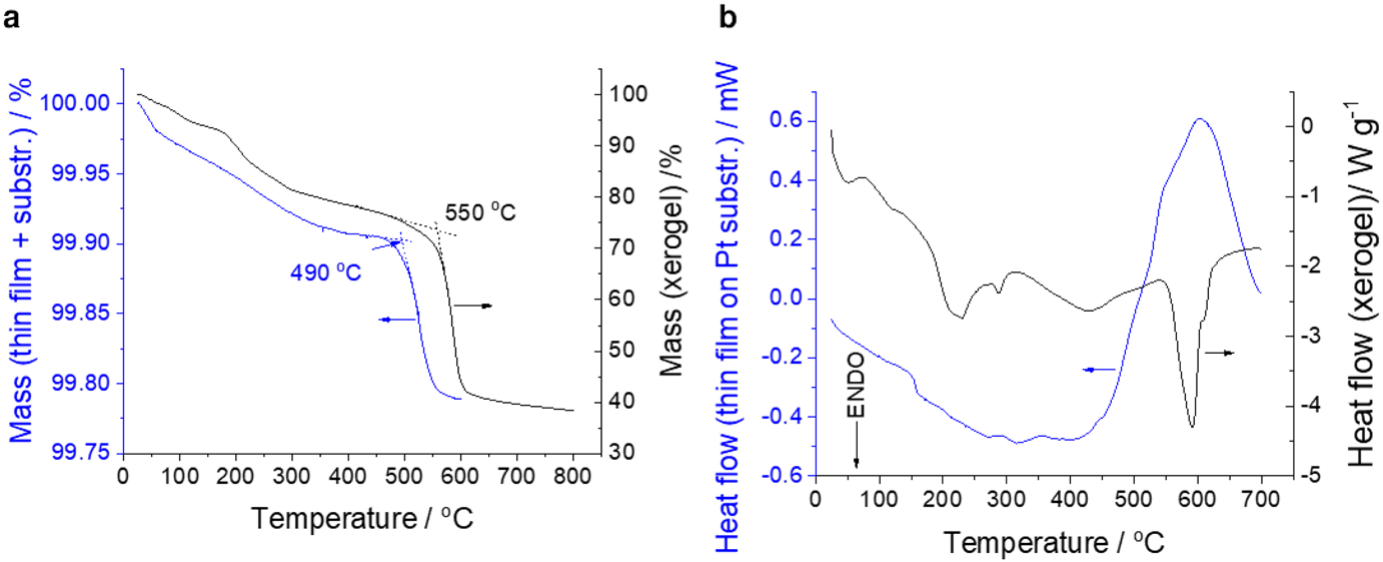
Comparison of TG curves (**a**) and DSC curves (**b**) of a thin film and corresponding xerogel. For the TG measurement, the films were mounted on microscope coverslips and, after drying, cut into small pieces that could be placed in a crucible. Xerogels with initial mass of about 5 mg were analyzed using the same temperature protocol. The heating rate was 5 K/min. Platinum crucibles of 150 µL were used. During the measurements, the furnace was purged with air (100 mL/min). For DSC measurements, a thin film was applied to the platinum foil (0.7 × 0.7 cm^2^), dried, and placed directly on the thermocouple. The bare Pt foil served as a reference. Xerogels were weighed into 70-µL Pt pans; in this case, an empty pan served as reference. The same measurement conditions were used as for the TG measurements

For sol–gel-prepared titania thin films (where the sol was prepared by dissolution of TiCl_4_ previously acidified with H_2_SO_4_), thermal decomposition of sulfate groups began at 550 °C for the xerogel but at 490 °C for thin films, deposited on microscope cover glasses (Fig. 12) [156]. The thickness of the films was around 100 nm. The DSC curve obtained from the thin film deposited on a Pt substrate and then directly on a thermocouple (Fig. 11) was different in comparison with that for the xerogel. The former showed a pronounced exothermic effect in the temperature range corresponding to anatase crystallization, while the DSC curve of the xerogel showed an endothermic effect. The authors explained the lower decomposition temperature of the thin films on the basis of the following facts: (1) thin films with a thickness of some tenths of nanometers can be composed of nanoparticles with a large number of surface atoms, having high surface energy, and are therefore more reactive; therefore, thermal decomposition occurs at lower temperatures; (2) the influence of the substrate on the behavior and thus thermal stability of the thin film with regard to xerogel samples; (3) the low thickness of thin films, which enables fast diffusion of the evolved gases on the surface of the solid phase [156].

Thermal analysis using the complementary TG and DSC techniques can be applied to follow the crystallization process of chemically prepared thin films, while coupled TG–MS analysis of the corresponding xerogel provides a sufficient quantity of the evolved gaseous product to describe the course of the thermal decomposition. To confirm the changes that occur, additional diffraction and spectroscopic techniques can be used ex situ. A correct interpretation of the structural changes in the films can be achieved on the basis of the results of thermal analysis of the thin films themselves since their decomposition can occur at different temperatures with regard to xerogels.

### UV–Vis and IR Spectroscopic Methods

Photophysical properties of films are frequently estimated with the help of UV–Vis spectroscopy. The Tauc method [180] for estimating the bandgap energy of amorphous semiconductors using optical absorption spectra is most widely used. However, misapplication of the Tauc plot to determine the bandgap energy of semiconductors may lead to erroneous estimates. Ghobadi [181] used the derivation ineffective thickness method (DITM) to extract the optical bandgap energy and the type of optical transitions in nanostructured semiconductors. In this method, the optical bandgap is obtained independently from the thickness measurement and only requires measurement of the absorbance spectrum [181].

Infrared spectroscopy is widely used in studies of solar photoactive films. Far-IR (FIR) FTIR spectra can reveal morphological features of the films; For example, Miquelot et al. [182] used the shoulder at ca. 520 cm^−1^ of 350-nm-thick TiO_2_ films to identify voids among the crystallites [183].

Since the IR absorption frequency depends also on the interatomic distance, among other factors, it can provide supplementary proof regarding the fraction and quality of the different catalyst polymorphs present in the sample. In fact, in solar-active photocatalysts, the use of this technique is relatively frequent. For instance, it was used to reveal the evolution of BiO_6_, BiO_3_ and BiO compounds with increasing temperature leading to β-Bi_2_O_3_ phase at 450 °C for usage in removal of Methyl Orange in a continuous flow solar reactor [184]. The same principle can be extended further by using IR spectroscopy in transient mode, thus revealing the dynamics of molecular species in photoactive materials. Recently, researchers proposed a transient (lasting for a couple of hundred nanoseconds) structural change from hematite to maghemite in α-Fe_2_O_3_ thin films following light excitation [101]. The change was detected by a transient peak at 640 cm^−1^, suggesting a local distortion similar to maghemite following excitation. The longevity of the transient change correlated excellently with the photocurrent of the films under Xe lamp illumination, which is similar to that of sunlight, hence demonstrating the usefulness of the transient FTIR method for the characterization of thin films.

### Electrochemical Methods

Electrochemistry is the branch of physical chemistry that studies the interplay between electricity and chemistry. The goal is to measure electrical quantities such as current, potential, or charge and their relationship to chemical parameters. Electrochemical measurements have found a wide range of applications, including environmental monitoring, industrial quality control (corrosion control), and biomedical applications. A typical electrochemical experimental setup consists of an electrochemical cell (the system under investigation) and a potentiostat. The electrochemical cell can include two, three, or four electrodes. The most common form for the electrochemical cell has three electrodes: (1) the working electrode which is under investigation (for example, a glassy carbon electrode), (2) the counterelectrode (usually made of platinum, silver, etc.), which is necessary to close the electrical circuit, and (3) a reference electrode (for example, Ag/AgCl, calomel, etc.), which is used to determine the potential across the electrochemical interface accurately. To close the electrical circuit, the electrodes are immersed in a liquid (e.g., Na_2_SO_4_ [185, 186], a mixture of Na_2_S and Na_2_SO_3_ [187], KOH [185], KCl containing K_3_[Fe(CN)_6_] [188], etc.) which is the electrolyte. Conductive indium tin oxide (ITO) [187] or fluorine-doped tin oxide (FTO) [186] glasses are commonly used as substrates for the working electrodes. In some cases, also screen-printed electrodes are used [185, 188]. Screen-printed electrodes offer the benefit that the size of the whole electrochemical cell setup can be minimized, thus a small amount of electrolyte can be used. The investigated photocatalysts can be deposited in the form of thin films on the surface of the working electrode by using different deposition techniques. These techniques involve deposition by dipping of the working electrode substrates into a solution of a binder and the photocatalyst [185, 188] or by spraying/spreading of the binder/photocatalyst solution onto the electrode surface [186, 187]. Electrochemical impedance spectroscopy (EIS), cyclic voltammetry (CV), and transient photocurrent measurements are commonly used.

**Fig. 13:**
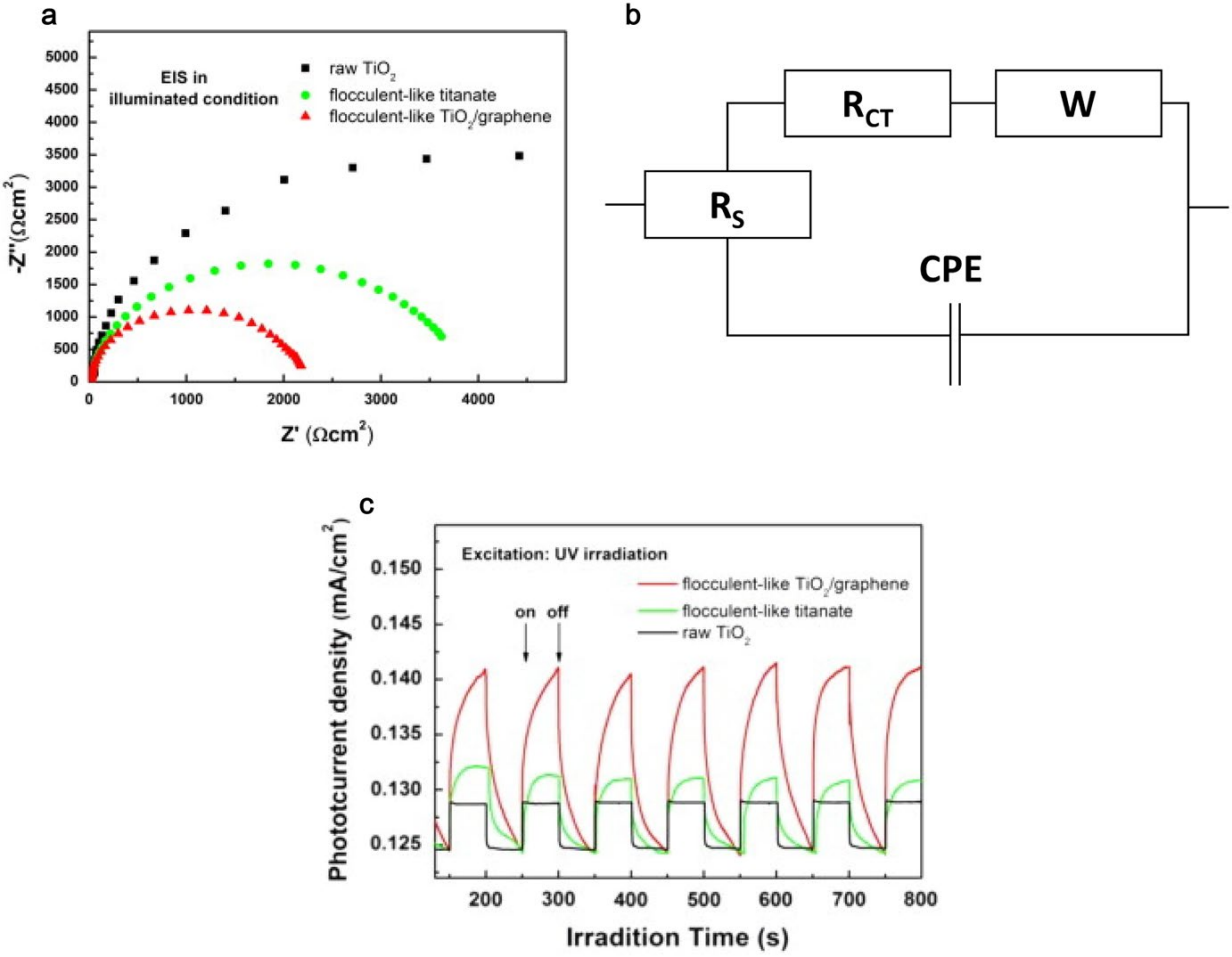
**a** EIS Nyquist plots of TiO_2_/graphene and raw TiO_2_ measured under UV-light irradiation [187]. **b** Electrochemical equivalent circuit used by Žerjav et al. [189] to fit the Nyquist plots of TiO_2_ and TiO_2_ + Au photocatalysts, consisting of solution resistance (*R*_S_), charge-transfer resistance (*R*_CT_), Warburg impedance (*W*), and a constant-phase element (CPE). **c** The photocurrent density response of TiO_2_/graphene and raw TiO_2_ measured under intermittent UV-light illumination [187]

The promise of EIS is that, with a single experimental procedure encompassing a sufficiently broad range of frequencies, the influence of the governing physical and chemical phenomena can be isolated and distinguished, at a given applied potential. The fundamental approach of EIS methods is to apply a small-amplitude sinusoidal excitation signal to the system under investigation and measure the response. The graph of the real versus the imaginary part of the impedance is called the Nyquist plot (Fig. 13a). The intermediate-frequency response in a Nyquist plot is related to electron transfer and transfer at the electrode (catalyst surface)–electrolyte interface [104]. The diameters of the semicircles in the Nyquist plots reflect the charge-transfer process, with smaller diameters indicating lower charge-transfer resistance [83, 105]. Han et al. [187] employed EIS measurements (Fig. 13a) to examine the separation of photogenerated electron–hole pairs under UV-light illumination in thin films of TiO_2_/graphene nanocomposite deposited onto the working electrode by spreading a solution of PTEF and the prepared catalyst. The semicircle of the flocculent-like TiO_2_ in the plot became smaller with the introduction of graphene, illustrating a reduction of the resistance between the solid-state interface layer and the charge transfer on the surface. The semicircle of the flocculent-like TiO_2_ was smaller than that for raw TiO_2_, as a result of the high charge mobility of the flocculent-like TiO_2_. These EIS measurements show that, in the composite, charge recombination was suppressed and thus the lifetime of the carriers was prolonged. This leads to higher photocatalytic activity, which was confirmed by the results of photodegradation of Methyl Orange under UV illumination [187]. Žerjav et al. [189] investigated the separation of visible-light-generated charge carriers in composite TiO_2_ + Au films using EIS measurements and explained the obtained Nyquist plots on the basis of the electrochemical equivalent circuit (EEC, Fig. 13b) composed of the solution resistance (*R*_S_), charge-transfer resistance (*R*_CT_), Warburg impedance (*W*), and a constant-phase element (CPE). The *R*_CT_ in the EEC indicates the value of the charge-transfer resistance at the electrode (thin film of photocatalyst)–electrolyte interface [190]. It is not surprising that, under visible-light illumination, bare TiO_2_ expressed the highest *R*_CT_ value as only illumination with UV light can trigger the generation of charge carriers in TiO_2_. On the other hand, the *R*_CT_ values of the TiO_2_ + Au composite were found to be significantly lower in comparison with those of TiO_2_ because, owing to the plasmonic properties of Au, the composite can generate charge carriers also under visible-light illumination. Here, we should point out that the configuration of the electrochemical equivalent circuit used to explain/fit the results of EIS measurements depends on the configuration of the electrochemical experimental setup applied [191]. One must be aware of this effect when comparing EIS results from different researchers.

**Fig. 14:**
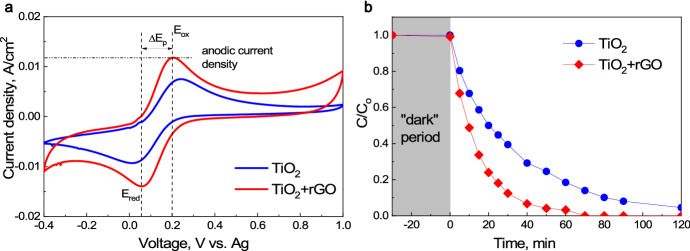
**a** Cyclic voltammograms recorded for TiO_2_ and TiO_2_ + rGO measured in 0.1 M KCl solution containing 1 mM K_3_[Fe(CN)_6_] as redox probe at scanning rate of 50 mV/s. **b** Photocatalytic degradation of BPA in presence of TiO_2_ and TiO_2_ + rGO irradiated with UVA light for 120 min [188]

Cyclic voltammetry (CV) is a widely used technique for obtaining qualitative information about electrochemical reactions. In cyclic voltammetry, linear scanning of the potential of a stationary working electrode with a triangular waveform is used. Depending on the information sought, single or multiple cycles may be used. During the potential sweep, the potentiostat measures the current resulting from the applied potential. The resulting current–potential plot is called a cyclic voltammogram. The cyclic voltammogram is a complicated, time-dependent function of a large number of physical and chemical parameters. Žerjav et al. [188] combined TiO_2_ with reduced graphene oxide (rGO) and analyzed the electronic properties of the prepared materials by using CV measurements performed in KCl solution containing K_3_[Fe(CN)_6_] as a redox probe. In Fig. 14a, the anodic and cathodic peaks for each sample are clearly visible. The peaks at positive potentials on the anodic (forward) sweep at around 0.2 V versus Ag represent the oxidation (*E*_ox_) of ferrocyanide to ferricyanide with loss of one electron. The cathodic peak at about 0.05 V versus Ag represents the reduction (*E*_red_) of ferricyanide to ferrocyanide [192]. Low peak-to-peak separation (Δ*E*_p_ = *E*_red_ − *E*_ox_) is related to fast electron-transfer kinetics. The reported Δ*E*_p_ values clearly indicate (Fig. 14a) that, in comparison with pure TiO_2_, the electron transfer kinetics is accelerated in TiO_2_ + rGO. The amount of electrons transferred is higher in the TiO_2_ + rGO composite as the anodic current density of TiO_2_ + rGO is higher than that of pure TiO_2_. In heterogeneous photocatalytic reactions, higher amounts of electrons and accelerated electron transfer increase the photocatalytic activity of the catalyst. The results of the photocatalytic oxidation of water-dissolved endocrine-disrupting component bisphenol A (BPA) under UV illumination in Fig. 13b completely correlate with the results of the CV measurements, indicating improved charge separation and migration in the TiO_2_ + rGO catalyst due to the junction between TiO_2_ and rGO.

Transient photocurrent measurements, where the photocurrent density on the working electrode is measured versus the time of illumination/shadowing of the working electrode, can be used to investigate the excitation and transfer of photogenerated charge carriers in the examined photoctalyst. Han et al. [187] measured the photocurrent response of thin TiO_2_/graphene nanocomposite films on ITO glass under intermittent UV-light illumination (Fig. 13c). The photocurrent density of the TiO_2_/graphene nanocomposite was twice that of raw TiO_2_. This increase in the photocurrent density was attributed to the introduction of graphene, which could enhance the charge transfer owing to its excellent electrical conductivity for storing and shuttling electrons [187]. The results of the photocurrent measurements were in good agreement with those from EIS measurements (Fig. 13a), which also revealed that the introduction of graphene suppressed the rate of charge-carrier recombination and prolonged the lifetime of the charge carriers in comparison with raw TiO_2_.

## Conclusions and Outlook

Although the use of coatings is advocated because of their practical advantages, their application has not yet gained wide acceptance in the scientific community. The main reasons for this are: (1) the production of stable and visually satisfactory coatings requires a great deal of effort and knowledge, (2) it is difficult to characterize such films, and (3) the culprits of poisoning of photocatalytic films are not well known and are extremely difficult to study. This requires special and more cumbersome experimental conditions. Finally, it seems that films are used more frequently at higher stages of technological maturity, i.e., at research stages closer to commercialization and thus, in a sense, further from the interest of the scientific community.

It is imperative to address the above challenges in the future, as solar-powered photocatalytic films are essential for sustainable air and water purification, water splitting, hydrogen evolution, CO_2_ reduction, and sustainable chemical production. Most of the solar radiation reaching the Earth is visible (42%) and infrared light (49%). Only a small fraction of ultraviolet radiation reaches the surface (8%). This is an important fact because science needs to focus on materials that are photocatalytically active in all three regions of the solar spectrum. It is worth noting that the strategies used to design the structures of photocatalytic films are not significantly different from those used for heterogeneous photocatalysts in powder form (e.g., doping, surface deformation, semiconductor coupling, and defect design), and the scientific community has a great deal of knowledge in this regard. In general, nanostructuring of semiconductor layers means moving from single- or polycrystalline materials exhibiting a classical planar semiconductor–liquid–gas interface to nano- or even microscale geometries, i.e., nanoparticles, nanorods, nanowires, nanocones, nanoflowers, etc., forming a three-dimensional interface with a large surface area. Among the various factors affecting the performance of films under solar irradiation, two of them are obviously the mentioned structure of the films and the film thickness. We have shown that the optimum film thickness is often around 1 µm, which is commonly attributed to the sufficient absorption and scattering at this value. However, in certain situations/applications, a lower film thickness may be advantageous, so optimization is recommended for each individual study. There are several deposition methods for thin-film fabrication. Deposition methods with promising scalability in industry are spin coating and dip coating, We have shown that different characterization methods (e.g., thermal analysis, UV–Vis and IR spectroscopy, electrochemistry, etc.) can be applied to characterize the properties of solar-active coatings, depending on which properties are the key factor for the photocatalytic activity in the solar-active systems under study and are therefore the subject of research. However, adhesion is one of the most important parameters for evaluating the quality of photocatalytic coatings.

This review describes for the scientific community the engineering of already known materials (e.g., TiO_2_ and ZnO) as well as the discovery of emerging, novel materials that are sunlight active. Some promising candidates include porous organic polymers (POPs) [193, 194] and their corresponding group of covalent organic frameworks (COFs) [195, 196], among others. In this respect, novel mechanisms that take better advantage of the incoming irradiation in all parts of the solar spectrum are also appearing, e.g., photothermal catalysis [197]. Both aspects could find great application in the future, also in the field of solar-driven photocatalytic films.
